# Dietary n-3 long-chain polyunsaturated fatty acids upregulate energy dissipating metabolic pathways conveying anti-obesogenic effects in mice

**DOI:** 10.1186/s12986-018-0291-x

**Published:** 2018-09-26

**Authors:** Stefanie Worsch, Mathias Heikenwalder, Hans Hauner, Bernhard L. Bader

**Affiliations:** 1 Else Kroener-Fresenius-Center for Nutritional Medicine, Chair of Nutritional Medicine, Technical University of Munich, Freising, Germany; 2 ZIEL - Institute for Food and Health, Nutritional Medicine Unit, Technical University of Munich, Freising, Germany; 3 Else Kroener-Fresenius-Center for Nutritional Medicine, University Hospital Klinikum rechts der Isar, Uptown München-Campus D, Technical University of Munich, Georg-Brauchle-Ring 60/62, 80992 Munich, Germany; 4 Division of Chronic Inflammation and Cancer, German Cancer Research Center (DKFZ), Heidelberg, Germany

**Keywords:** n-3 long-chain polyunsaturated fatty acids, n-6/n-3 PUFA ratio, High-fat diet, Diet-induced obesity, Brown adipose tissue, Energy metabolism, Thermogenesis, Futile cycling, Fibroblast growth factor 21 (FGF21), ß-oxidation

## Abstract

**Background:**

We previously reported on the anti-obesogenic and anti-inflammatory effects associated with n-3 long-chain polyunsaturated fatty acids (LCPUFA) in our diet-induced obesity (DIO) mouse model. Two isocaloric high-fat diets (HFDs; 48 kJ% fat), HFD (HF) and n-3 LCPUFA-enriched HFD (HF/n-3), and a control diet (C; 13 kJ% fat) were used. The underlying mechanisms however have largely remained unclear. Here, we assessed whether the reduced fat mass reflected n-3 LCPUFA-induced expression changes in lipid metabolism of the intestine, liver, and interscapular brown adipose tissue (iBAT), as well as increased iBAT thermogenic capacity.

**Methods:**

For HF/n-3, saturated and monounsaturated fatty acids were partially substituted by n-3 LCPUFA eicosapentaenoic acid and docosahexaenoic acid to achieve a balanced n-6/n-3 PUFA ratio (0.84) compared to the unbalanced ratios of HF (13.5) and C (9.85). Intestine, liver and iBAT from male C57BL/6 J mice, fed defined soybean/palm oil-based diets for 12 weeks, were further analysed. Gene and protein expression analyses, immunohistochemistry and correlation analyses for metabolic interactions were performed.

**Results:**

Compared to HF and C, our analyses suggest significantly diminished de novo lipogenesis (DNL) and/or increased hepatic and intestinal fatty acid oxidation (ω-oxidation and peroxisomal β-oxidation) in HF/n-3 mice. For iBAT, the thermogenic potential was enhanced upon HF/n-3 consistent with upregulated expression for uncoupling protein-1 and genes involved in mitochondrial biogenesis. In addition, a higher capacity for the supply and oxidation of fatty acids was observed and expression and correlation analyses indicated a coordinated regulation of energy metabolism and futile cycling of triacylglycerol (TAG). Moreover, HF/n-3 significantly increased the number of anti-inflammatory macrophages and eosinophils and significantly enhanced the levels of activated AMP-activated protein kinase α (AMPKα), peroxisome proliferator-activated receptor α (PPARα) and fibroblast growth factor 21 (FGF21).

**Conclusions:**

Our data suggest that by targeting transcriptional regulatory pathways, AMPKα, and FGF21 as potential mediators, HF/n-3 activated less efficient pathways for energy production, such as peroxisomal β-oxidation, increased ATP consumption upon the induction of futile cycling of TAG, and additionally increased the thermogenic and oxidative potential of iBAT. Therefore, we consider n-3 LCPUFA as the potent inducer for upregulating energy dissipating metabolic pathways conveying anti-obesogenic effects in mice.

**Electronic supplementary material:**

The online version of this article (10.1186/s12986-018-0291-x) contains supplementary material, which is available to authorized users.

## Background

Obesity represents a major risk factor for metabolic disorders, especially cardiovascular diseases, type 2 diabetes and fatty liver disease [1, 2]. Overconsumption of a ‘Western diet’ - foods consisting high levels of sugars and fats - is a major cause of the epidemic prevalence of obesity [3]. The dietary fat quality influences obesity development, since fatty acids individually modulate the expression levels of several genes and transcriptional factors that are involved in lipid and energy metabolism regulation [4]. It is generally believed that, besides saturated fatty acids (SFA), the n-6/n-3 polyunsaturated fatty acids (PUFA) ratio, which has steadily increased in Western diets, is associated with increasing obesity and metabolic disorders [5, 6], whereas, in particular, animal studies reported that n-3 long-chain polyunsaturated fatty acids (LCPUFA) and/or their metabolites have anti-obesogenic and anti-inflammatory potential, and monounsaturated fatty acids (MUFA) are also metabolically benefical [7]. However, mixed results as well as the lack of consistency and findings across human studies [8] make it difficult to confirm these observations in humans. In particular, the underlying mechanisms are still not well understood and data from tissues of human studies are very limited. Mouse studies have shown that n-6 PUFA-derived lipid mediators act pro-inflammatory, whereas n-3 PUFA-derived lipid mediators confer anti-inflammatory and pro-resolving actions in inflamed adipose tissue by eliciting macrophage polarization toward a M2-like phenotype [9] and/or by the reduction of crown-like structures (CLS) rich in inflammatory M1 macrophages in obese mice [10]. Moreover, n-3 PUFA can improve impaired metabolism in obesity by modulating main metabolic pathways in adipose tissue, liver and small intestine in mice [11–14]. In addition, brown adipose tissue (BAT) expansion and/or activation and “browning” of white adipose tissue (WAT) can result in increased energy expenditure in mice by uncoupled respiration (thermogenesis), and targeting BAT and/or browning of WAT might prove a potential strategy for the treatment of obesity and related metabolic disorders in humans [15]. In this context, the activation of the sympathetic nervous system by cold or overfeeding induces the thermogenic activity of BAT by the activation of uncoupling protein 1 (UCP1) and by increasing the supply and oxidation of metabolic substrate to sustain thermogenesis [16]. Various other factors and signaling pathways, such as the hepatic factor fibroblast growth factor 21 (FGF21) or bile acid-mediated activation of the G protein-coupled bile acid receptor (TGR5) can activate BAT or induce browning of WAT, and alternatively activated M2 macrophages can control BAT thermogenesis via local release of catecholamines [17–19]. In recent years, both the cold-induced thermogenesis and the diet-induced thermogenesis (DIT) have been studied increasingly also at molecular level. Mechanisms of DIT include uncoupling of mitochondrial respiration through UCP1, but also the activation of futile metabolic cycles, whereby PUFA are particularly effective in recruiting brown/beige adipocytes [20]. Emerging evidence suggested that a fish oil diet, rich in n-3 PUFA, promotes BAT thermogenesis [21, 22], although the underlying mechanisms remain poorly described [23]. The observed phenotypes and the impact of interventions from diet-induced obesity (DIO) mouse models however are sometimes controversial across studies due to the lack of a standardized defined high-fat diet (HFD) for modeling DIO. This makes it also difficult to compare the data of the studies. The importance of source and type of dietary fat for the regulation of metabolic pathways has been shown by nutrigenomic research demonstrating the differential impact of dietary fatty acids and triglycerides on gene regulation via peroxisome proliferator-activated receptor α (PPARα) [24, 25]. Thus, we previously established a DIO mouse model using defined soybean/palm oil-based diets [26]. Compared to the control diet C (13 kJ% fat), isocaloric HFDs (48 kJ% fat) - HF, and n-3 LCPUFA-enriched HFD (HF/n-3) - were characterised by differential fat quality and unbalanced n-6/n-3 PUFA for C (9.85) and HF (13.5) and balanced n-6/n-3 PUFA for HF/n-3 (0.84). Interestingly, after 12-week feeding, HF/n-3 mice compared to HF mice showed reduced body weight and fat mass, lower hepatic triacylglycerol (TAG) and plasma non-esterified fatty acids (NEFA) levels, although they ingested more and excreted less energy than HF mice. In addition, differential metabolic and inflammatory responsiveness to diets of visceral adipose tissues and increased UCP1 mRNA levels in interscapular brown adipose tissue (iBAT) were observed [26]. To explain these observed anti-obesogenic effects, we hypothesised that compared to HF mice, HF/n-3 mice have the potential for (1) altered lipid handling, either by diminished de novo lipogenesis (DNL) and/or increased fatty acid oxidation in the intestine, liver, and iBAT, and (2) increased DIT in iBAT. Consequently, the aim of our study was to analyse the expression changes in key genes and proteins involved in these metabolic and inflammatory processes and their regulation with a main focus on iBAT.

## Methods

### Mice and diets

Organs and tissues were used from mice of our previous feeding study as described in detail in Ludwig et al. [26]. In brief, for the 12-week feeding trial, the 8-week old male C57BL/6J mice, housed under specific-pathogen-free conditions and with ad libitum access to food and water, were randomly assigned to three dietary groups (*n* = 12 mice/group): control diet (C) with 13 kJ% fat and the isocaloric HFDs with 48 kJ% fat, HF and n-3 LCPUFA-enriched HFD (HF/n-3). All diets (C, cat. no. S5745-E720; HF, cat. no. S5745-E722; HF/n-3, cat. no. S5745-E725) were purified experimental diets manufactured as pellets by Ssniff Spezialdiäten (Soest, Germany). Diet compositions are shown in Table 1 and diet fatty acid patterns in Additional file 1: Table S1. All experimental procedures were conducted according to the German guidelines on animal care and handling and were approved (permission no: 55.2–1–54-2532-113-11) by the Governmental district of Upper Bavaria (Oberbayern), Germany.

**Table 1:** Diet compositions

Nutrient	C	HF	HF/n-3
Lipids (%)	**5.01**	**25.04**	**25.02**
Soybean oil	5.00	5.00	5.00
Palm oil	–	20.00	11.25
EPAX 1050 TG (DHA/EPA)	–	–	8.75
Cholesterol	0.013	0.038	0.021
Carbohydrates (%)	**63.39**	**43.39**	**43.39**
Corn starch	47.79	27.79	27.79
Protein (%)	**24.00**	**24.00**	**24.00**
Antioxidants (%)	**0.03**	**0.03**	**0.03**
Others (%)	**7.60**	**7.60**	**7.60**
Metabolizable energy (MJ/kg)	**15.50**	**19.80**	**19.80**
Fat (kJ%)	13.00	48.00	48.00
Carbohydrates (kJ%)	64.00	34.00	34.00
Protein (kJ%)	23.00	18.00	18.00
Fatty acid composition (%)			
Ʃ SFA	16.55	42.66	29.83
Ʃ MUFA	25.95	37.14	29.05
Ʃ PUFA	56.90	19.70	40.12
Ʃ n-6 PUFA	50.85	18.07	18.19
Ʃ n-3 PUFA	5.16	1.34	21.68
n-6/n-3 PUFA ratio	9.85	13.50	0.84
Metabolizable energy, fat and fatty acids (kJ%)	**13**	**48**	**48**
Ʃ SFA	2.2	20.5	14.3
Ʃ MUFA	3.4	17.8	14.0
Ʃ PUFA	3.6	9.5	19.3
Ʃ n-6 PUFA	3.2	8.7	8.7
Ʃ n-3 PUFA	0.3	0.6	10.4

Shown are weight% or energy% macronutrients and other ingredients in different diets and the fat content is further characterised. Nutrient composition was kept constant across different diets, except for corn starch and fat components. Metabolizable energy was calculated according to Atwater in MJ/kg (provided by Ssniff Spezialdiäten) *C* control diet (Cat. no.: S5745-E720), *DHA* docosahexaenoic acid, *EPA* eicosapentaenoic acid, *HF* high-fat diet (Cat. no.: S5745-E722), *HF/n-3* n-3 long-chain polyunsaturated fatty acid-enriched high-fat diet (Cat. no.: S5745-E725), *MUFA* monounsaturated fatty acids, *PUFA* polyunsaturated fatty acids, *SFA* saturated fatty acids

### Sample collection, total RNA, and protein extraction

At the end of the feeding period, animals were euthanized in the postprandial state. Amongst other tissues, liver, intestinal mucosa [mucosal scrapings of the upper and lower small intestine (USI and LSI)] and iBAT were sampled, weighed, snap frozen in liquid nitrogen and stored at − 80 °C or further processed for histological procedures. RNA and protein extractions were performed as previously described in detail [26]. In brief, after tissue homogenization with Qiazol lysis reagent total RNA was extracted with chloroform, purified using the mRNeasy mini kit (Qiagen, Hilden, Germany) and further processed (on-column DNase I digestion, quantity and quality measurement). Extraction of total protein from tissues was performed in the presence of proteinase and phosphatase inhibitors (Sigma-Aldrich, Taufkirchen; Roche, Mannheim, Germany). Protein concentration was determined by bicinchoninic acid method (Pierce Thermo Scientific, Bonn, Germany).

### Quantitative real-time PCR

Expression of specific target genes was evaluated with real-time quantitative polymerase chain reaction (RT-qPCR). For each sample, 10 ng of extracted RNA was used per reaction using the QuantiTect quantitative, real-time one-step RT-PCR kit (Qiagen, Hilden, Germany), following the supplier’s protocol. Primers and commercially available primer assay are listed in the Additional file 1: Table S2, S3. RT-qPCR was performed using SYBR Green I dye and a Mastercycler ep *realplex4* S (Eppendorf, Hamburg, Germany), as described earlier [26]. The ΔΔCq method was applied for analysis [27]. To normalise the data the following genes were used as invariant controls. iBAT: cyclophilin B (Cypb) and heat shock protein 90 (cytosolic) and class B member 1 (Hsp90αb1); liver: hypoxanthine-guanine phosphoribosyltransferase1 (Hprt1) and β-actin (Actb); USI: glyceraldehyde-3-phosphate-dehydrogenase (Gapdh), Actb and Hprt1; LSI: Cypb and Hsp90αb1. For all groups, data were expressed as means ± standard error of the means (SEM) relative to control samples.

### Triacylglycerol content in iBAT

TAG were extracted as follows. Grounded tissues were homogenised in HB-buffer [10 mM NaH_2_PO_4_, 1 mM EDTA, 1% polyoxyethylen (10) tridecyl ether]. Supernatents were collected after centrifugation (4 °C; 23,100 g; 15 min) and incubated for 5 min at 70 °C. After incubation on ice for 5 min and centrifugation (4 °C; 23,100 g; 15 min) the TAG concentrations were determined in the samples using a serum triglyceride determination kit (Sigma-Aldrich, Taufkirchen, Germany), following the manufacturer’s instructions. Results were normalised to total weight and total protein content of the tissue (determined by bicinchoninic acid method).

### Western blot analysis

Equal amounts of protein (30–80 μg/lane) were resolved by a 7.5, 10 or 12.5% SDS-PAGE. Subsequently, proteins were transferred to a nitrocellulose membrane using a Tankblot Eco-Mini system (Biometra, Göttingen, Germany) and further processed as described previously [26]. Primary antibodies for antigen detection are listed in the Additional file 1: Table S4. IRDye800CW-conjugated goat anti-rabbit secondary antibody or IRDye680RD-conjugated donkey anti-mouse secondary antibody were applied for visual detection using the Odyssey Infrared Detection system (LI-COR Biotechnology, Bad Homburg, Germany). β-ACTIN or HSP90 expression served as invariant controls.

Recruitment of iBAT was calculated as UCP1 content in total iBAT: [UCP1 expression * dilution factor for protein content * (total iBAT in mg / weighed portion of iBAT for protein isolation in mg)] (normalised values were used).

Relative UCP1 capacity index was calculated as follows: [(relative protein expression * total iBAT tissue (mg))/body mass (mg)] * 100%.

### Histological analysis

For immunohistochemistry, 6-μm-thick paraffin tissue sections (*n* = 5/group, in duplicates) were dewaxed and stained with specific primary antibodies (Additional file 1: Table S4), and further processed as previously described [26]. Photographs at × 400 magnification are used for composite and close-ups from representative cluster of macrophages (MCL), similar to CLS, which were recently also described in BAT [28], were taken at × 630 magnification. MCL was described as at least three positively stained cells surrounding a lipid droplets containing cell. MCL density was defined as number of MCL per total section area (mm^2^). Hematoxylin and eosin-stained paraffin sections were used to analyse iBAT tissue morphology.

### Statistical analysis

All data were expressed as means ± SEM. Statistical analyses were performed using Prism 5 (GraphPad Software). Differences were considered statistically significant with * *p* < 0.05, ** *p* < 0.01, *** *p* < 0.001 compared to control group or high-fat groups. For normal distributed samples, one-way ANOVA was used to detect differences between dietary groups with Tukey’s post-test to identify significant differences. In case of no normal distribution or *n* < 5, Kruskal-Wallis test and Dunn’s post-test were applied. In addition, outliers were detected by using Grubb’s test (GraphPad Software) and excluded from analysis. For immunohistochemical analyses, the detection of positive-stained cells were made in duplicates and mean values were used for statistical analyses. Pearson correlation coefficient (r) was employed to investigate correlations (two-tailed test) and tests for linear trend were calculated by using linear regression models.

## Results

### HFDs differentially upregulate genes involved in peroxisomal β-oxidation and ω-oxidation in the intestine and in the liver

**Fig. 1:**
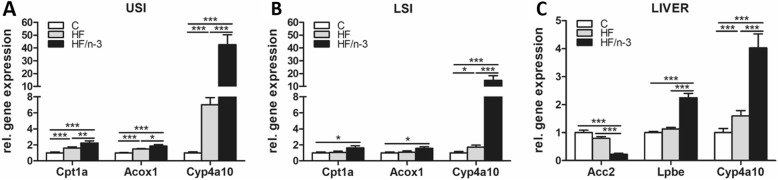
Metabolic gene expression in upper (USI) and lower small intestine (LSI) and the liver. All data are means ± SEM and were collected from mice after 12-week feeding either control (C), HFD (HF), or n-3 long-chain polyunsaturated fatty acid (LCPUFA)-enriched HFD (HF/n-3). **a**, **b** Gene expression of carnitine palmitoyl transferase 1a (Cpt1a), acyl-coenzyme A oxidase 1 (Acox1) and cytochrome P450, family 4, subfamily a, polypeptide 10 (Cyp4a10) representing proteins involved in mitochondrial and peroxisomal β-oxidation as well as ω-oxidation in USI (**a**; *n* = 7–11) and in LSI (**b**; *n* = 8–12). **c** Expression of genes operating in de novo lipogenesis, mitochondrial β-oxidation as well as ω-oxidation in liver (*n* = 10–12): Acetyl-CoA c arboxylase 2 (Acc2), enoyl-Coenzyme A hydratase (Lpbe) and Cyp4a10. RT-qPCR data were normalised to Hprt1, Gapdh and Actb (USI), Cypb and Hsp90αb1 (LSI) or Hprt1 and Actb (liver), and calculated relative to controls. *p < 0.05, **p < 0.01, ***p < 0.001, significant differences compared to control or between groups as indicated

mRNA levels of proteins involved in mitochondrial and peroxisomal β-oxidation and in ω-oxidation were analysed separately in the upper and lower small intestine (USI and LSI). The expression levels of these genes were significantly increased in the USI for both high-fat groups, whereas significantly higher levels were detected for HF/n-3 than for HF (Fig. 1a). For LSI, these genes were only significantly upregulated upon HF/n-3, except for cytochrome P450, family 4, subfamily a, polypeptide 10 (CYP4a10; ω-oxidation). This gene showed the highest fold changes, and was also significantly higher expressed for HF than for C (Fig. 1b). For liver, CYP4a10 expression was also significantly increased for both HFDs compared to control. However, the difference (2.53-fold) between HF/n-3 and HF was lower as the intestinal expression (USI: 6.08-fold; LSI: 8.63-fold). In addition, the mRNA expression for enoyl-Coenzyme A hydratase (LPBE; peroxisomal β-oxidation) was significantly higher and for acetyl-CoA carboxylase 2 (ACC2; DNL) was significantly lower in the HF/n-3 group than for control and HF group (Fig. 1c).

### Downregulation of hepatic DNL upon HFDs

**Fig. 2:**
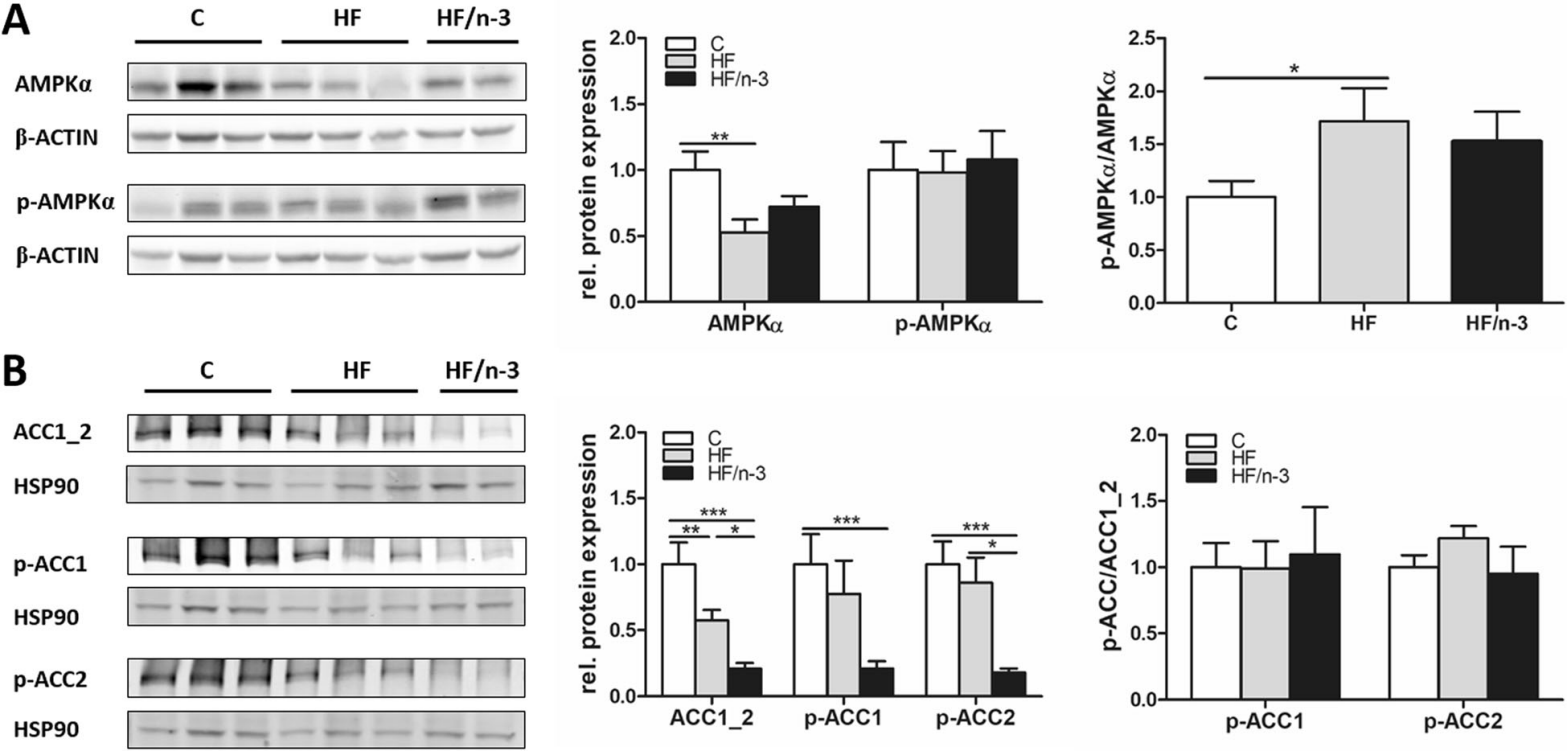
Protein expression of enzymes operating in hepatic de novo lipogenesis. All data are means ± SEM and were collected from mice after 12-week feeding either control (**c**), HFD (HF), or n-3 long-chain polyunsaturated fatty acid (LCPUFA)-enriched HFD (HF/n-3). **a**, **b** Western blot analyses of (**a**) AMP-activated protein kinase α (AMPKα) and (**b**) acetyl-CoA carboxylases (ACC1_2) and their representative phosphorylations in liver (*n* = 5–6). Separate gels were run for quantification of total AMPKα (62 kDa) and p-AMPKα (Thr172; 62 kDa) and ACC1_2 (265 kDa and 280 kDa), p-ACC1 (Ser79; 257 kDa) and p-ACC2 (Ser219/Ser221; 275–280 kDa), respectively. Shown are the protein expression levels relative to controls and the ratios for the phosphorylated forms to total protein expression levels (normalised values were used). β-ACTIN (42 kDa) or HSP90 (90 kDa) was used for protein normalisation. *p < 0.05, **p < 0.01, ***p < 0.001, significant differences compared to control or between groups as indicated

Hepatic DNL was further characterised by examining protein expression of cellular energy sensor AMP-activated protein kinase α (AMPKα, Fig. 2a) and ACC1_2 (Fig. 2b), since activated AMPK (pThr172-AMPKα) can phosphorylate ACC1 and 2 on pSer79-ACC1 and pSer219/Ser221-ACC2, respectively, and inhibit DNL [29]. Compared to controls, in HF mice total AMPKα was significantly decreased, whereas the p-AMPKα/AMPKα ratio was significantly increased. A similar tendency for p-AMPKα/AMPKα ratio was also observed upon HF/n-3. Moreover, hepatic levels for total ACC1_2 were significantly reduced in both high-fat groups, with a stronger downregulation for HF/n-3 than for HF. Levels for p79-ACC1 were only significantly reduced for HF/n-3 compared to control, whereas pSer219/Ser221-ACC2 levels were significantly lower for HF/n-3 than for control and HF. Interestingly, the ratios pSer79-ACC1/ACC1_2 and pSer219/pSer221-ACC2/ACC1_2 were not significantly different between the groups. Thus, our data suggest that hepatic DNL is diminished upon chronic feeding of HF and HF/n-3 by down-regulation of ACC1_2 protein expression and exlude the possibility that ACC1 and 2 are inactivated by a sharp increase of phosphorylated ACC1 and 2 levels upon activated AMPK (pThr172-AMPKα).

### HFDs increase iBAT mass and TAG content

As previously reported, chronic feeding of HFDs significantly increased iBAT mass compared to control and regardless of fat quality [26], whereas TAG content in total iBAT as well as amount of TAG/gram of protein showed only a significant increase for the HF mice (Table 2). Noteworthy, for HF compared to control, in iBAT the protein content (protein amount/iBAT mass) was significantly lower.

**Table 2:** iBAT mass, triacylglyerol and protein content

Mass	C			HF			HF/n-3		
iBAT (mg)	179.3	±	21.2	312	±	15.5^a^	246.8	±	18.2^a, b^
Subgroup (*n* = 5)									
TAG (mg)/g iBAT	92.6	±	9.3	118.1	±	13.7	118.4	±	12.7
TAG (mg) in total iBAT	20.7	±	1.6	40.7	±	6.9^a^	32.1	±	5.6
Protein (mg)/g iBAT	49.2	±	3.6	37.0	±	1.3^a^	45.4	±	2.0
Protein (mg) in total iBAT	11.2	±	1.1	12.5	±	0.7	11.9	±	1.2
TAG (mg)/protein (mg)	1.9	±	0.1	3.3	±	0.5^a^	2.6	±	0.2

All data are means ± SEM and were collected from mice after 12-week feeding either control (C), HFD (HF), or n-3 long-chain polyunsaturated fatty acid (LCPUFA)-enriched HFD (HF/n-3). Index letters a and b indicate a significant difference (*p* < 0.05) compared to C and HF group, respectively

*iBAT* interscapular brown adipose tissue, *TAG* triacylglycerol

### Increased thermogenic capacity in iBAT upon HF/n-3

**Fig. 3:**
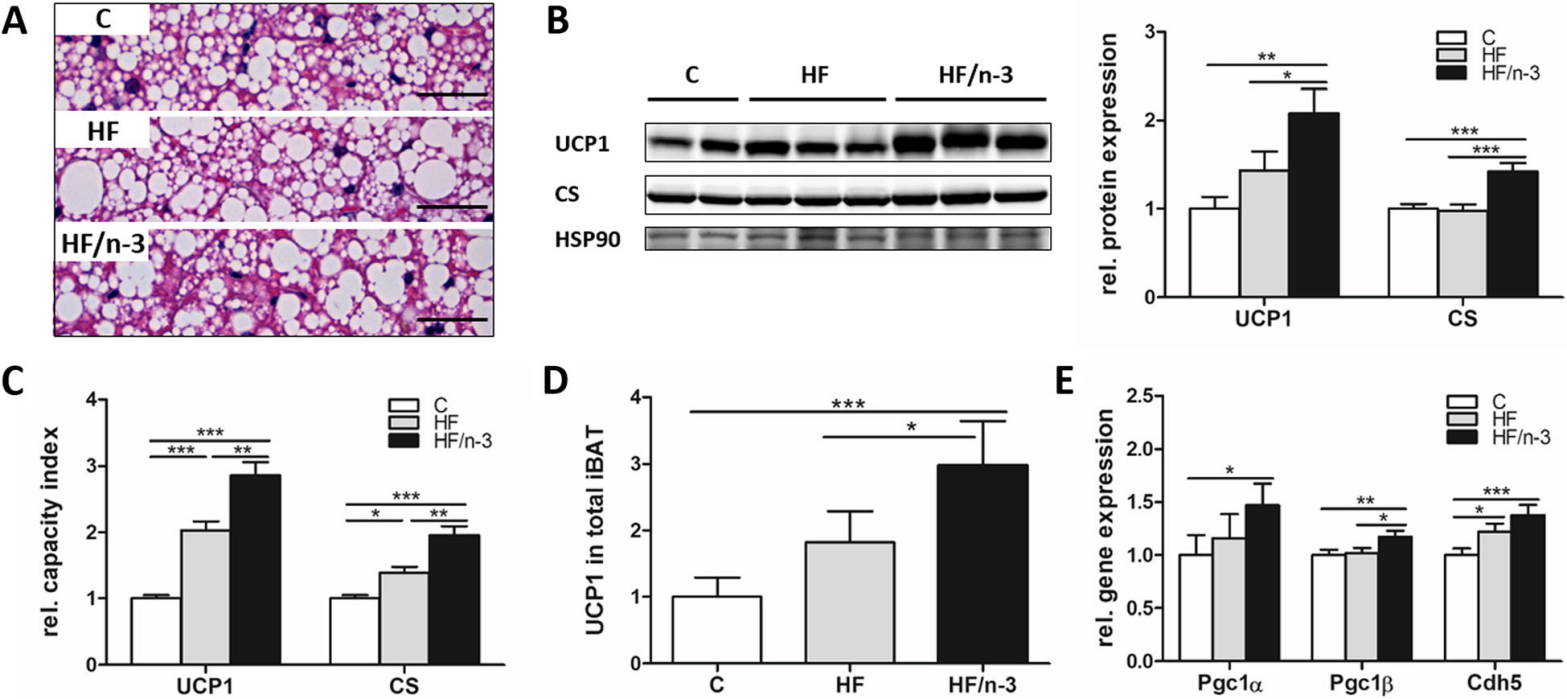
Thermogenic capacity of iBAT. All data are means ± SEM and were collected from mice after 12-week feeding either control (C), HFD (HF), or n-3 long-chain polyunsaturated fatty acid (LCPUFA)-enriched HFD (HF/n-3). **a** Representative microscopic images of hematoxylin and eosin-stained paraffin sections of iBAT. Scale bars indicate 50 μm. **b** Western blot analyses of the mitochondrial proteins uncoupling protein 1 (UCP1; 32 kDa) and citrate synthase (CS; 52 kDa) in iBAT (*n* = 5–6). Shown are the protein expression levels relative to controls. HSP90 (90 kDa) was used for protein normalisation. **c** Relative capacity index of UCP1 and CS (normalised values were used, see Methods). **d** UCP1 content in total iBAT (normalised values were used, see Methods). **e** Gene expression levels of peroxisome proliferator-activated receptor γ coactivator 1-α (Pgc1α) and -β (Pgc1β), genes involved in mitochondrial biogenesis, and of the endothelial marker cadherin 5 (Cdh5) in iBAT (*n* = 9–12). RT-qPCR data were normalised to CypB and Hsp90αb1, and calculated relative to controls. *p < 0.05, **p < 0.01, ***p < 0.001, significant differences compared to control or between groups as indicated

Brown adipocyte number, the mitochondrial density, and the amount of UCP1 define thermogenic potential of iBAT, but adipose tissue vascularisation makes also its contribution [16]. Analysing iBAT morphology, surprisingly, for both high-fat diets increased lipid droplet size was observed (Fig. 3a). Importantly, however, levels of the mitochondrial proteins UCP1 and citrate synthase (CS) were significantly elevated for HF/n-3 compared to control and HF, whereas they were not significantly different between HF and C (Fig. 3b). However, considering differences in iBAT and body mass between dietary groups in relation to UCP1 and CS expression, both protein levels were significantly higher for HF than for C, and for HF/n-3 they remained significantly elevated compared to HF and C (Fig. 3c). Finally, calculated UCP1 content for total iBAT (see Methods) and mRNA levels for the peroxisome proliferator-activated receptor γ coactivator 1-α and-β (PGC1A and PGC1B; [30]) were significant higher for HF/n-3 than for HF and control (Fig. 3d, e). These data indicate lower mitochondrial density for the HF mice compared to HF/n-3 mice. Furthermore, mRNA levels for the endothelial cell marker cadherin 5 (CDH5) were significantly elevated for both HFDs compared to control (Fig. 3e).

### HF/n-3 changes lipid and glucose metabolism in iBAT

**Fig. 4:**
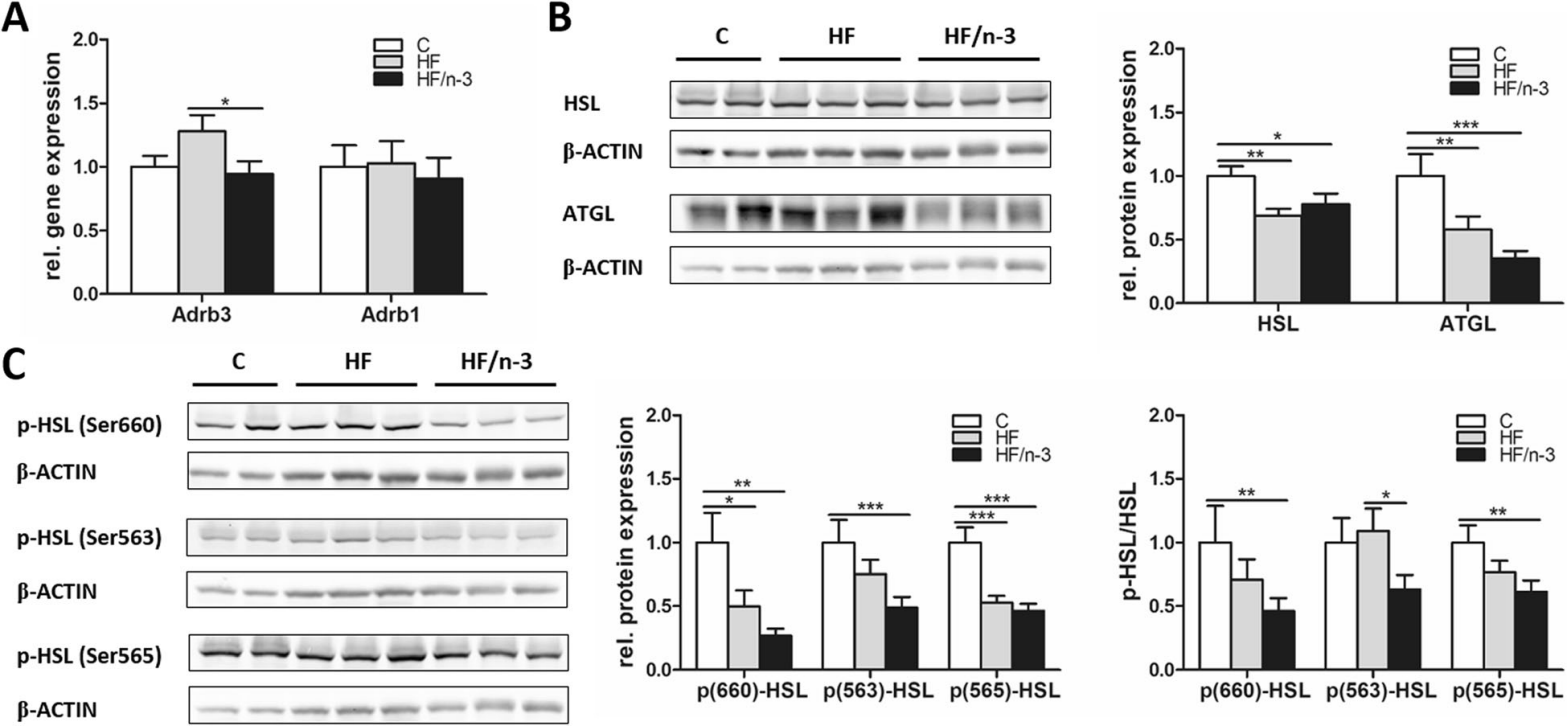
Gene and protein expression of markers involved in lipolysis in iBAT. All data are means ± SEM and were collected from mice after 12-week feeding either control (C), HFD (HF), or n-3 long-chain polyunsaturated fatty acid (LCPUFA)-enriched HFD (HF/n-3). **a** Gene expression data of the β-adrenergic receptors Adrb3 and Adrb1, which convey the signals from catecholamines to stimulate lipolysis, in iBAT (*n* = 10–12). RT-qPCR data were normalised to Cypb and Hsp90αb1, and calculated relative to controls. **b**, **c** Western blot analyses of (**b**) hormone sensitive lipase (HSL) and adipose triglyceride lipase (ATGL) and (**c**) representative phosphorylations of HSL in iBAT (*n* = 5–6). Separate gels were run for quantification of total HSL (81 and 83 kDa) and p-HSL (Ser660), p-HSL (Ser563) and p-HSL (Ser565; each 81 and 83 kDa), respectively, whereby the blotting membrane of p-HSL (Ser565) was reincubated for the detection of ATGL (54 kDa). Shown are the protein expression levels relative to controls and, for HSL, the ratios for the phosphorylated forms to total protein expression levels (normalised values were used). β-ACTIN (42 kDa) was used for protein normalisation. *p < 0.05, **p < 0.01, ***p < 0.001, significant differences compared to control or between groups as indicated

Activation of the sympathetic nervous system induces the thermogenic activity of BAT by activating UCP1 and increasing the supply and oxidation of metabolic substrates to sustain thermogenesis [16]. To examine whether the observed increased lipid storage in iBAT upon HFDs can be explained by potential differences in lipid handling, we measured the expression of genes operating in fatty acid uptake and oxidation, TAG storage, lipolysis and DNL, as well as glucose uptake or glycolysis (Table 3). In addition, as shown in Fig. 4, lipolysis and its stimulation by catecholamine, which activates beta-adrenergic receptors (ADRB), were further assessed by expression analyses of ADRB, hormone sensitive lipase (HSL), adipose triglyceride lipase (ATGL) and lipase activity control. Phosphorylation of HSL on Ser660 or Ser563 activates lipolysis, whereas Ser565 phosphorylation inhibits lipolysis by downregulating Ser563 phosphorylation [31].

**Table 3:**
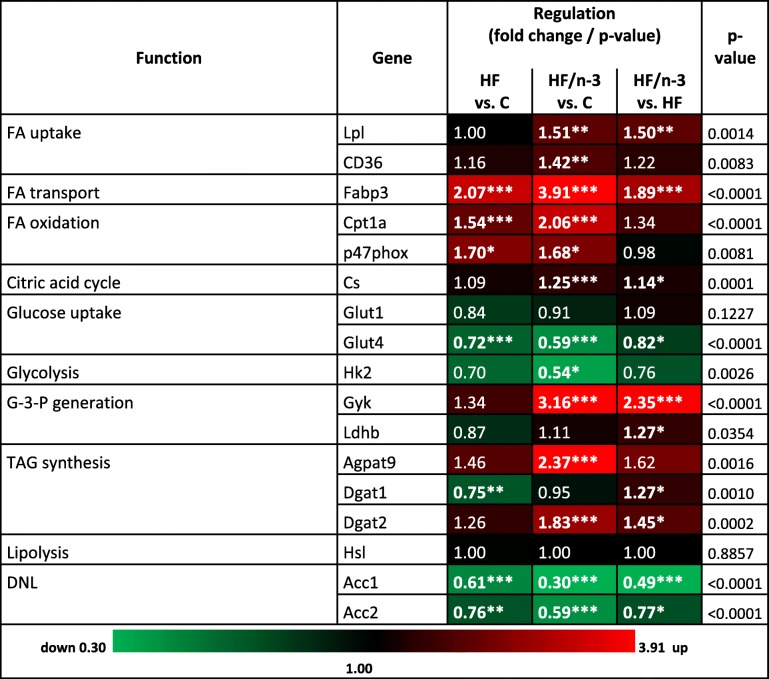
Summary of metabolic gene expression changes upon HF and HF/n-3 in iBAT

Expression regulations of genes with different metabolic functions in iBAT between HF and control (C) diet, HF/n-3 and C or HF/n-3 and HF. Shown are fold changes with corresponding *p*-values as indicated (significant differences: *p < 0.05, **p < 0.01, ***p < 0.001). Statistically significant fold changes are shown in bold. The up- and downregulated gene expression is illustrated as heatmap to facilitate the visualisation of gene expression pattern

*DNL* de novo lipogenesis, *FA* fatty acid, *G-3-P* glycerol-3-phosphate, *TAG* triacylglycerol

A heat-map-like presentation of data for the targeted mRNA expression analyses is shown in Table 3. Lipoprotein lipase (LPL) and fatty acid translocase (CD36) mRNA were significantly increased for HF/n-3 compared to control, and LPL levels were even significantly higher for HF/n-3 than for HF indicating increased lipid uptake in iBAT. Expression for fatty acid binding protein 3 (FABP3), carnitine palmitoyl transferase 1a (CPT1a) and the NAD(P)H oxidase subunit p47PHOX was significantly upregulated for HFDs compared to controls, whereby the significantly higher FABP3 level for HF/n-3 represents the second highest change between HFDs. In addition, the CS expression was also significantly elevated for HF/n-3 compared to control and HF. Glucose transporter 4 (GLUT4) mRNA was significantly reduced for HF/n-3 and HF compared to control, and GLUT4 levels were even lower for HF/n-3 than for HF. GLUT1 mRNA expression showed no regulation and hexokinase 2 (HK2) levels were significantly decreased upon HF/n-3 compared to control. With regard to lipid storage, for HF/n-3, significantly elevated mRNA levels for glycerol kinase (GYK) and lactate dehydrogenase (LDHB) were detected compared to HF, whereas for GYK (highest change between HFDs), 1-acylglycerol-3-phosphate O-acyltransferase 9 (AGPAT9) and diglyceride acyltransferase 2 (DGAT2) levels were higher for HF/n-3 than for control. DGAT2 mRNA was even significantly elevated upon HF/n-3 compared to HF, whereas DGAT1 levels were significantly lower for HF than for control. Significantly decreased ACC1 and ACC2 expression was detected for both HFDs compared to control, and levels were substantially lower for HF/n-3 than for HF.

Lipolysis in iBAT was further explored as shown in Fig. 4. A significant upregulation of ADRB3 mRNA levels was found for HF compared to HF/n-3, whereas ADRB1 was not regulated (Fig. 4a). HSL mRNA levels were not changed (Table 3). However, compared to controls, protein expression for HSL and ATGL as well as for phosphorylated HSL p660-HSL and p565-HSL was significantly reduced upon HF and HF/n-3 (Fig. 4b, c), whereas p563-HSL was only significantly lower for HF/n-3 but not for HF. For the ratios p660-HSL/HSL and p565-HSL/HSL, they were significantly lower for HF/n-3 than for control, whereas the ratio p563-HSL/HSL for HF/n-3 was significantly reduced compared to HF (Fig. 4c). Taken together, although the data indicated that the free fatty acids (FFA) supply for mitochondria seems more likely be sustained by increased lipid uptake than TAG lipolysis in HF/n-3 mice, correlation analyses showed significant correlations between the gene expression levels of DGAT2 and HSL (*r* = 0.6377, *p* = 0.0473) in HF/n-3 mice pointing to a dynamic interplay between TAG synthesis and hydrolysis upon HF/n-3.

### HF/n-3 upregulates markers of thermogenic activation and energy metabolism in iBAT

To further identify regulatory mechanism involved in the promotion of iBAT activity and energy expenditure, we focused on catecholamine-mediated activation of β-adrenergic receptor signaling, and adrenergic-independent brown fat activator pathways [17] - TGR5–Iodothyronine 5′-deiodinase (DIO2) and G protein-coupled receptor (GPR120)-FGF21 [18, 32] as well as signaling pathway interaction of eosinophils and macrophages [19, 33] and corresponding enzyme tyrosine hydroxylase (TH; [19, 34]). Moreover, the metabolic hormone FGF21 [35] and key metabolic regulators - the transcription factors PPARα and γ2 [36, 37] and transducin-like enhancer of split 3 (TLE3; [38]), leptin (LEP) and AMPKα [29] - were analysed in iBAT.

**Fig. 5:**
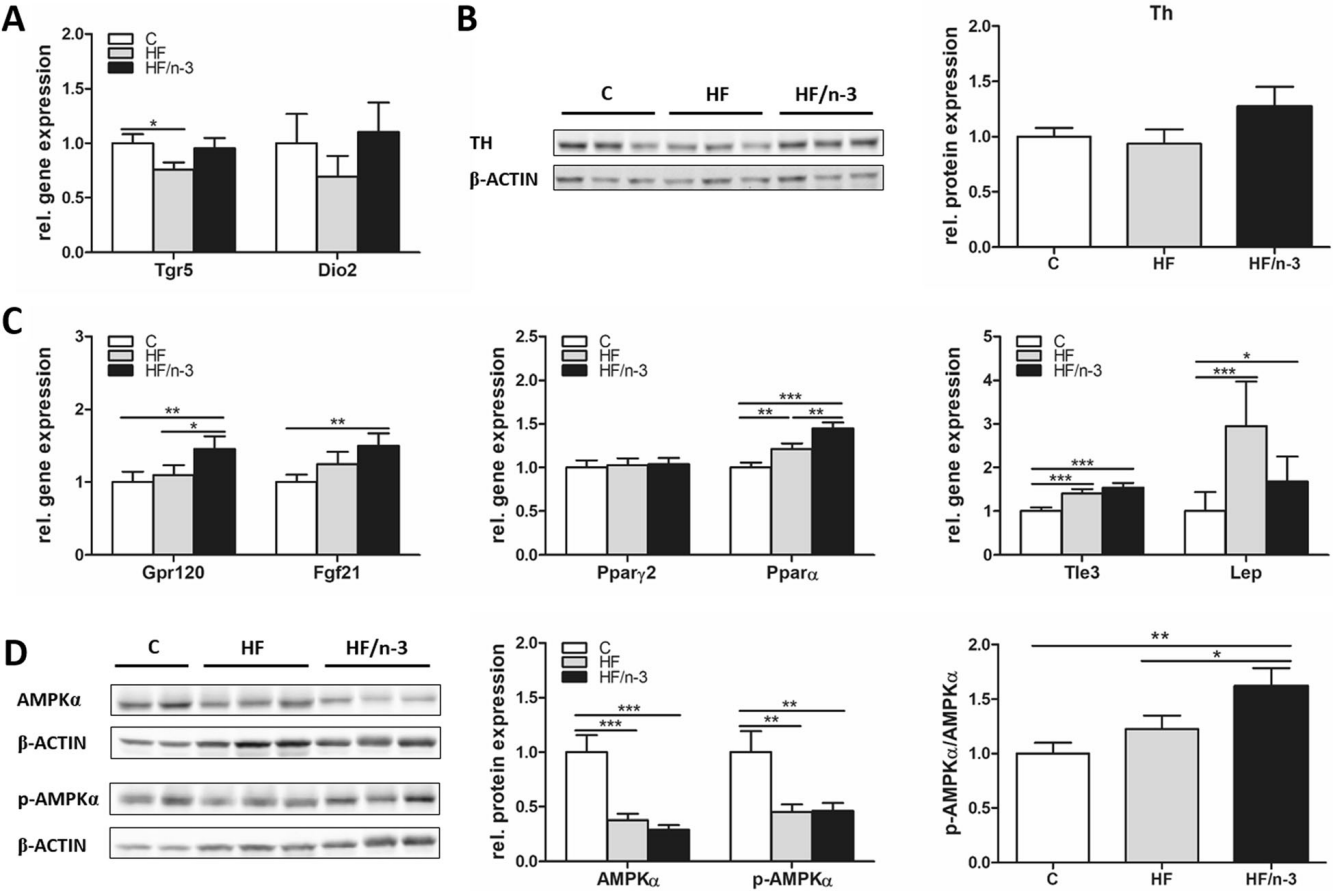
Gene expression of proteins associated with thermogenic activation and energy metabolism in iBAT. All data are means ± SEM and were collected from mice after 12-week feeding either control (C), HFD (HF), or n-3 long-chain polyunsaturated fatty acid (LCPUFA)-enriched HFD (HF/n-3). **a**, **b** Expression of genes and proteins associated with thermogenic activation in iBAT. **a** Shown are mRNA levels of proteins involved in a signaling pathway for local generation of triiodothyronine (*n* = 10–12). **b** Western blot data of tyrosine hydroxylase (TH; 62 kDa), a catecholamine-synthesizing enzyme in iBAT (*n* = 5–6). **c**, **d** Expression data of metabolic regulators in iBAT. **c** Shown are the gene expression levels (*n* = 9–12) of the G protein-coupled receptor (GPR120), the fibroblast growth factor 21 (FGF21), the transcription factors peroxisome proliferator-activated receptor (PPAR) γ2 and α, the transcriptional co-repressor of BAT-selective gene expression transducin-like enhancer of split 3 (Tle3), and leptin (Lep). **d** Western blot analyses of AMPKα (62 kDa) and p-AMPKα (Thr172; 62 kDa) in iBAT (*n* = 5–6). For proteins, the expression levels relative to controls and the ratios for the phosphorylated forms to total protein expression levels are shown (normalised values were used). β-ACTIN (42 kDa) was used for protein normalisation. RT-qPCR data were normalised to Cypb and Hsp90αb1, and calculated relative to controls. *p < 0.05, **p < 0.01, ***p < 0.001, significant differences compared to control or between groups as indicated

In contrast to our finding for ADRB3, TGR5 mRNA levels were significantly reduced for HF compared to control in iBAT. DIO2 mRNA levels were not regulated (Fig. 5a) and TH protein expression was only slightly higher for HF/n-3 than for control (Fig. 5b). mRNA levels for GPR120, FGF21, PPARα, TLE3 and LEP were significantly elevated for HF/n-3 compared to controls, whereas only PPARα, TLE3 and LEP showed significant higher levels for HF than for controls. Expression levels of GPR120 and PPARα were even significantly higher for HF/n-3 than for HF, whereas PPARγ2 was not regulated (Fig. 5c). Finally, significantly reduced protein expression levels for total AMPKα and p-AMPKα were found in both HFD groups compared to controls. However, the ratio was only significantly increased for HF/n-3 compared to control and HF (Fig. 5d).

To further explain the different phenotypes of iBAT and the functional state of iBAT upon differential diets, we performed correlation analyses. Significant or nearly significant correlations were found in the HF/n-3 group between the mRNA levels of PPARα, GPR120 or FGF21 and UCP1, whereas for the HF group, a significant positive correlation was observed between the mRNA levels of FGF21 and UCP1, however, also negative correlations were found between the mRNA levels of TLE3 and UCP1 (Additional file 1: Table S5). Considering FGF21 as an upregulated downstream target upon PPAR activation [35] and as activator of several downstream signaling pathways including AMPKα [39], FGF21 might be involved in the observed positive regulation of energy metabolism in HF/n-3 mice. Significant correlations between the mRNA levels of FGF21 and ADRB1, CD36 or DGAT1 were measured within all groups (Additional file 1: Table S6A). Moreover, especially within the HF/n-3 group, significant or nearly significant correlations were observed between (1) the mRNA levels for ADRB1, CD36 and DGAT1 (Additional file 1: Table S6A), and (2) between ADRB1, CD36 and DGAT1 mRNA levels and mRNA levels of almost all other analysed enzymes involved in energy metabolism (Additional file 1: Table S6B).

### Increased macrophage and eosinophil marker expression in iBAT upon HF/n-3

**Fig. 6:**
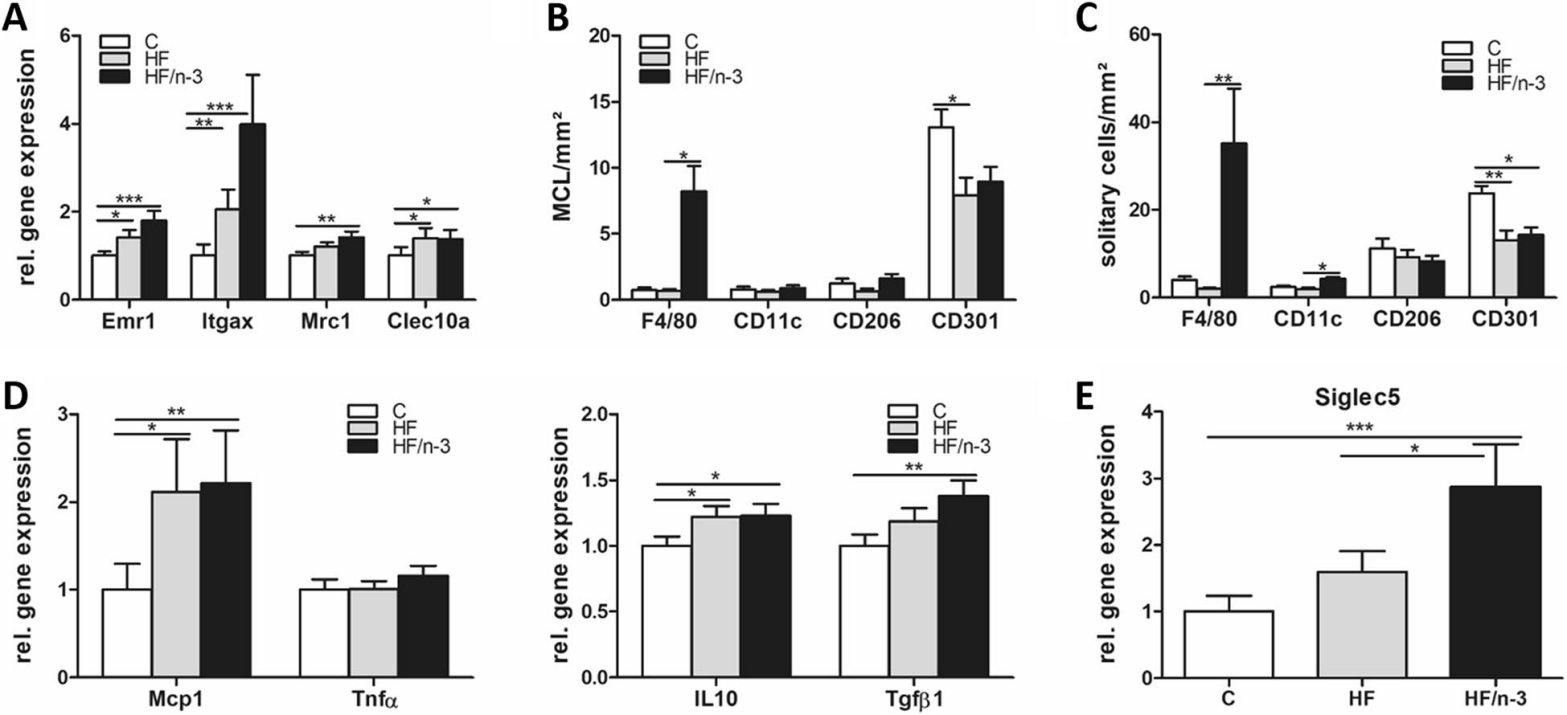
Inflammatory status of iBAT. All data are means ± SEM and were collected from mice after 12-week feeding either control (C), HFD (HF), or n-3 long-chain polyunsaturated fatty acid (LCPUFA)-enriched HFD (HF/n-3). **a** Gene expression data of macrophage markers in iBAT (*n* = 10–12): EGF-like module-containing mucin-like hormone receptor-like 1 (EMR1; F4/80), integrin αX (ITGAX; CD11c), mannose receptor C type 1 (MRC1; CD206) and C-type lectin domain family 10, member A (CLEC10a; CD301). **b**, **c** Quantification of cluster of macrophages (MCL) and solitary cells, detected by immunohistochemical analyses (images provided in Fig. 7), which are positive for F4/80, CD11c, CD206 or CD301 in iBAT (*n* = 4–5). Number of (**b**) positive-stained MCL and (**c**) positive-stained solitary cells is expressed as MCL or solitary cells per mm^2^ of tissue section. Gene expression data of (**d**) pro- or anti-inflammatory chemokines / cytokines and (**e**) an eosinophil-specific marker in iBAT (*n* = 7–12). RT-qPCR data were normalised to CypB and Hsp90αb1, and calculated relative to controls. *p < 0.05, **p < 0.01, ***p < 0.001, significant differences compared to control or between groups as indicated

**Fig. 7:**
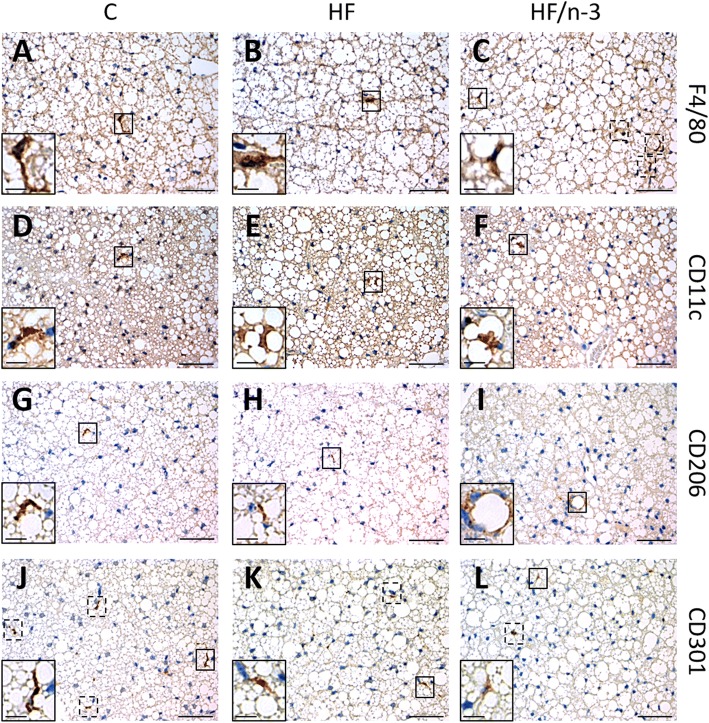
Immunohistochemical analysis of macrophage-associated M1 and M2 marker expression in clusters of macrophages (MCL) of iBAT. Shown are microscopic images of paraffin sections of iBAT from mice fed control (**a, d, g, j**), HF (**b, e, h, k**) or HF/n-3 diet (**c, f, i, l**) after immunohistochemical staining followed by counterstaining with hematoxylin. Data for F4/80 (**a, b, c**), CD11c (**d, e, f**), CD206 (**g, h, i**) or CD301 (**j, k, l**) are presented. Positive-stained cells in MCL surrounding lipid droplets are highlighted by frames with continuous or dashed line, respectively. Inserts of the images (lower left corner) show one representative structure of each image at higher magnification. Scale bars indicate 50 μm for the section and 20 μm for the insert

Given that certain immune cells might help to sustain adaptive thermogenesis [19, 33], whereas inflammation could downregulate the activity of iBAT [40], we examined immune cell infiltration and inflammation in iBAT. Adipose tissue macrophages were identified by the macrophage marker F4/80, encoded by mucin-like, hormone receptor-like sequence 1 (EMR1), and were classified according to their state of activation/polarization into the pro-inflammatory M1 macrophages and anti-inflammatory M2 macrophages. Integrin alpha X (ITGAX), mannose receptor, C type 1 (MRC1), and C-type lectin domain family 10, member A (CLEC10a), are known to encode CD11c (marker for M1 macrophage subset), CD206 and CD301 (markers for M2 macrophage subset), respectively [19, 41]. In iBAT, the gene expression levels of these markers (Fig. 6a) as well as their immunohistochemical detection (Fig. 7), and quantification of clusters of accumulated macrophages (MCL) surrounding adipocytes (Fig. 6b), in analogy to CLS in WAT, or as solitary cells (Fig. 6c) were examined. Moreover, the mRNA levels of pro- and anti-inflammatory markers (Fig. 6d) and sialic acid binding Ig-like lectin 5 (SIGLEC5; Fig. 6e) as eosinophil-specific marker [33] were determined.

Significantly higher mRNA levels for EMR1, but also for ITGAX and CLEC10a were detected upon both HFDs compared to control, whereas MRC1 level was only significantly increased upon HF/n-3 (Fig. 6a). Comparing immunohistochemical analyses of iBAT to visceral white adipose tissues, accumulated macrophages in iBAT form clusters without obvious CLS morphology as described in visceral adipose tissues [26], nevertheless CLS were described recently also in BAT [28]. Thus, we defined the accumulated macrophages in iBAT as cluster of macrophages (MCL). As shown in Fig. 6b, surprisingly, for all groups the CD301-positive stained MCL were more frequent than MCL stained positively for other applied macrophage markers, whereby CD301-positive stained MCL were significantly less upon HF compared to control. However, the number of CD11c- or CD206-positive-stained MCL was not different between diets. Notably, the most obvious changes were observed for F4/80-positive MCL, which were significantly more frequent upon HF/n-3 than upon HF. The results of the solitary cell counts were similar to those of the positive-stained MCL, with more F4/80-positive and CD11c-positive solitary cells upon HF/n-3 than HF, but the number of CD301-positive solitary cells was significantly downregulated in both high-fat groups compared to control group (Fig. 6c).

Besides that, the gene expression level of monocyte chemoattractant protein-1 (MCP1) was significantly higher for HFDs than control, whereas the pro-inflammatory marker tumor necrosis factors α (TNFα) showed no regulation. However, mRNA levels of the anti-inflammatory markers interleukin-10 (IL-10) and transforming growth factor β1 (TGFβ1) were significantly higher for HF/n-3 than for controls but upon HF only IL-10 levels were significantly elevated compared to control (Fig. 6d).

Given the involvement of M2 macrophages and eosinophils in thermogenesis, the mRNA abundance of the eosinophil-specific marker SIGLEC5 was determined and a correlation-based approach was applied to examine a possible correlation between the abundance of immune cells and the thermogenic marker UCP1. Notably, the mRNA level of SIGLEC5 was significantly enhanced for the HF/n-3 mice compared to control and HF mice (Fig. 6e) and, within the HF/n-3 group, SIGLEC5 was significantly correlated with the gene expression levels of EMR1, MCR1 and CLEC10a, and even UCP1 (Additional file 1: Table S8). In addition, significant correlations were also found between the gene expression levels of GPR120 and EMR1, CLEC10a or SIGLEC5 (Additional file 1: Table S8).

## Discussion

In the current study we assessed the previously observed anti-obesogenic effects of chronic HF/n-3 feeding [26] based on our hypothesis that HF/n-3 induces the expression of genes involved in energy dissipating metabolic pathways and/or thermogenesis resulting in increased energy expenditure and reduced fat mass in mice.

The main findings from our analyses of the intestine, liver and iBAT suggest diminished DNL, enhanced combustion of fatty acids and more coordinated regulation of energy metabolism upon HF/n-3 compared to HF. In particular, HF/n-3 activates less efficient pathways, such as ω-oxidation and peroxisomal β-oxidation, for energy production in the intestine and the liver and increases ATP consumption upon the induction of a TAG / FFA cycle in iBAT. In addition, iBAT from HF/n-3 mice manifest increased thermogenic and oxidative potential. To our knowledge the induction of a TAG/FFA cycle in iBAT tissue is shown the first time by our study. Finally, we characterised transcriptional regulatory pathways and AMPKα as targets for possible underlying mechanisms of n-3 LCPUFA, whereby FGF21 might mediate the metabolic effects of HF/n-3 in iBAT, and the increase in rather anti-inflammatory macrophages and eosinophils can be associated with lipid buffering and even thermogenesis in iBAT.

### Fatty acid oxidation and DNL

Previous in-vivo studies already proposed that n-3 LCPUFA can reduce HFD-induced obesity and hepatic fat accumulation by targeting transcriptional regulatory networks and regulating genes involved in hepatic [13] and intestinal [14] lipid metabolism. Our findings are in line with these reports. HF/n-3 upregulated PPARα target genes involved in fatty acid oxidation, whereby the ω-oxidation gene CYP4a10 showed the highest fold change. Furthermore, the prominent activation of ω-oxidation and peroxisomal β-oxidation is consistent with dietary n-3 LCPUFA enrichment and with previous findings from fish oil-based HFDs [42]. Notably, since peroxisomal β-oxidation itself generates heat instead of ATP due to lack of an electron transport system [43], the enhanced peroxisomal fatty acids turnover upon HF/n-3 may partly explain our data on higher energy expenditure and the prevention of excessive hepatic lipid deposition in HF/n-3 mice [26]. Surprisingly, hepatic CPT1a was not induced upon HF/n-3 [26], questioning whether PPARα-modulated carnitine acyltransferase is the rate limiting step of mitochondrial β-oxidation in circumstances where long-chain fatty acids are preferentially channeled into peroxisomes for oxidation [44]. Moreover, given the role of malonly-CoA, the product of ACC2, and of AMPK in the control of CPT1 [29], our data do not support an obvious role of the AMPK-ACC2-CPT1 axis in providing a flux control over mitochondrial β-oxidation. The lower hepatic ACC1 and ACC2 levels upon HFDs in our study and also in a study using beef tallow-based HFD [45] indicate reduced hepatic DNL and may reflect a long-term adaptation to HFDs, although the extent of the expression changes might depend on PUFA, fat and carbohydrate content of the diet [46]. Moreover, metabolic improvements upon FGF21 administration were recently associated with reduced expression of ACC1 and ACC2 in liver, reducing lipogenesis and enhancing lipid oxidation independent of AMPK-dependent phosphorylation of ACC [47, 48].

### Energy expenditure and thermogenesis in iBAT

Increased iBAT mass, UCP1 content, and mitochondrial and vascular density found in iBAT from HF/n-3 mice resemble features of cold-induced iBAT activation [16] and are in line with the report of Bargut et al. [23] showing increased thermogenesis in iBAT upon fish oil-based HFD. Most importantly, although increased iBAT mass was detected for both HFDs, only HF/n-3 mice showed increased levels for UCP1, CS, PGC1α, and PGC1β in iBAT. In contrast, HF mice however had lower protein content and expressed elevated TLE3 levels in iBAT, known to antagonise thermogenesis [38]. These data suggest higher mitochondrial densities and thermogenic capacity in iBAT upon HF/n-3, whereas, the increased iBAT mass of HF mice may be related to lipid accumulation due to the lower oxidative potential or dysfunctional lipid metabolism. Furthermore, Chau et al. indicated that FGF21 regulates mitochondrial activity and enhances oxidative capacity through an AMPK-SIRT1-PGC1α-dependent mechanism in adipocytes [39].

### Futile cycling of TAG in iBAT

The presented data indicate for the first time, that besides UCP1 protein expression a second thermogenic pathway that relies on futile cycling of TAG was induced upon HF/n-3 in iBAT. Recent studies already suggested for WAT the involvement of a futile substrate cycle based on TAG lipolysis and energy demanding re-esterification as an UCP1 independent anti-obesity effect of n-3 LCPUFA [12, 49]. Moreover, it was suggested that FGF21 corrects obesity in mice partly by promoting futile cycling in adipose tissue [47]. Notably, the TAG/FFA substrate cycling in adipose tissues creates an efficient buffer system that provides fatty acids in time of metabolic need, thus allowing sufficient fatty acid flux without non-physiological increase in cellular NEFA [49]. It should be noted that long-chain acyl-CoA themselves can modulate fatty acid supply via feedback inhibition of lipolysis [50]. For HF/n-3 mice, our data indicated that the supply of FFA for mitochondria seems more likely be sustained by increased lipid uptake (LPL and CD36) than lipolysis of TAG, but they also indicated subsequent efficient fatty acid oxidation (CPT1a, PPARα, FABP3 [51]), and ultimately enhanced lipid turnover, as well as a balanced energy metabolism (Table S6B). Moreover, our indication of HF/n-3-induced futile cycling of TAG in iBAT is supported by the observed significant correlations between lipolysis (HSL) and fatty acid re-esterification (DGAT2) and measured upregulation of GYK expression [52] upon HF/n-3. Furthermore, our expression data are consistent with an increase in fatty acid uptake (LPL and CD36) and storage (GYK and LDHB), TAG synthesis (DGAT1, DGAT2, AGPAT9), as well as a balanced energy metabolism.

### Sympathetic activity for increased energy expenditure in BAT

Considering the importance of sympathetic activity for increased energy expenditure in BAT, our data do not a priori show reduced sympathetic activity upon HF/n-3, a condition that could finally prevent increased energy expenditure, despite of enhanced UCP1 levels. Our findings are therefore not consistent with the observed effect of a PPARγ agonist on BAT sympathetic activity described by Festuccia et al. [37]. However, our data on the adrenergic-independent brown fat activator pathway GPR120-FGF21 [18, 32] point to an activated state upon HF/n-3. This data together with the increased TH protein level and the higher abundance of eosinophils and type 2 macrophages indicate a higher iBAT sympathetic nerve activity and thermogenic function in HF/n-3 mice. This is consistent with reports showing beneficial effects of n-3 LCPUFA on the autonomic nervous system [53] and of immune cells on brown adipocyte activity upon cold [19, 33]. However, the latter was questioned recently by Fischer et al. [54]. Nevertheless, for the HF/n-3 group, our data point to a connection between alternatively activated macrophages, eosinophils and the expression of thermogenic genes regulated via GPR120 signaling in iBAT. Furthermore, both the higher macrophage abundance and the elevated directed lipid uptake in iBAT upon HF/n-3 could have contributed the previously observed lower plasma NEFA levels in HF/n-3 mice [26].

### PPARs, pathways and FGF21

We observed the activation of PPARα and AMPKα as potential targets in HF/n-3 iBAT, whereby the HF/n-3-induced increase in FGF21 and immune cells might mediate changes in the expression of enzymes related to energy metabolism and/or be involved in the regulation of iBAT activity, respectively [19, 32, 33, 39, 47, 48]. As noted above, the mere enhanced UCP1 levels upon PPARy agonist cannot enhance energy expenditure [37]. However, the induction of PPARα can contribute to thermogenic activation of BAT by coordinately regulating lipid catabolism and thermogenic gene expression via induction of PGC1α [36]. Our data indicated this effect in iBAT from HF/n-3 mice, showing that PPARα activation participates in UCP1 expression. Moreover, the observed elevated levels for GPR120 and FGF21 upon HF/n-3, implicate GRP120 to exert a dual role [32, 55], connecting inflammation and metabolism in iBAT. Finally, our findings suggest that n-3 LCPUFA exert their favorable effects counteracting on obesity by targeting distinct pathways, such as PPARα and AMPKα in iBAT, but also in liver and small intestine, thereby increasing energy expenditure, whereby FGF21 as downstream target of PPARs reflects the activation of PPARα by n-3 PUFA and the activation of several downstream signaling pathways including AMPKα [35, 39].

### Study limitations and the translation of data in mice to humans

There are some limitations of our study which should be considered. In general, although major parts of the lipid metabolism appear to be conserved between mice and humans, differences exist regarding the regulation of triglyceride levels via apolipoproteins such as APOA5 [56] or the target genes of PPARα, the master regulator of hepatic lipid metabolism [57]. Moreover, similarities and differences in BAT between mice and humans relating to metabolism and thermogenesis as well as their underlying mechanisms of regulation are still not well understood. These limitations should be taken into account when extrapolating from mice to humans findings on metabolic disease processes such as hypertriglyceridemia, fatty liver and obesity or cardiovascular diseases.

Our observed expression changes are consistent with either n-3 LCPUFA-mediated induction and/or reduced n6/n3 PUFA ratios, however we can not completely ruled out, that reduced levels of SFA and/or MUFA in the HF/n-3 diet due to replacement with n-3 LCPUFA may also have contributed to these changes; considering SFA are likely more obesogenic than MUFA and PUFA, whereas unsaturated fat, MUFA and PUFA, appear to be more metabolically beneficial [58]. To counteract obesity by balancing the n-6/n-3 PUFA ratio, we used high levels of n-3 PUFA (10.4 kJ%), which are similar high as in other murine DIO models using fish-oil based diets (e.g. Bargut et al. [23]). However these doses should be considered with caution with regard to human dietary recommendations for adults (n-3 PUFA 0.5–2 kJ%; Additional file 1: Table S1) and clinical trials [59–61]. Moreover, the role of supplementation with LCPUFA considering the identification of population-related genetic variants in the LCPUFA biosynthetic pathway [62] is not clear and needs more research efforts addressing gene-PUFA interactions relating differently the risk of diseases.

Regarding the translation of n-3 PUFA effects from mice to humans in the context of energy expenditure, Fan et al. [61] recently stated that the evidence on thermogenic function of n-3 PUFA in human clinical trials, mostly fish oil supplementation, remains inconclusive, whereas strong correlations between fish oil intake and reduced visceral adiposity were found. So far, this appears to originate from the confounding factors of study design, technical difficulties in BAT identification, as well as lack of access to human brown/beige fat tissue samples from clinical trials to study the underlying mechanisms of n-3 PUFA effects in BAT.

## Conclusion

Overall, manipulation of dietary fat quality by n-3 LCPUFA enrichment and balancing n-6/n-3 PUFA ratio seems a possible avenue for positive modulation of energy metabolism and immune cells in obese mice. Our findings are based on expression and biochemical data combined with correlation analyses, therefore in future experiments physiological and metabolic validations of our data on the induction of energy dissipating pathways by n-3 LCPUFA underlying the anti-obesogenic effects are necessary. Additionally, a more complete understanding of the various doses and individual effects of the n-3 LCPUFA EPA and DHA and/or their metabolites in physiological and pathological processes of white and brown adipose tissues in mice and humans is vitally necessary. Taken together, more future research will be ultimately required before translating discoveries from mouse models, clinical trials, and target pathways into recommendations for human intake of n-3 LCPUFA as preventive strategy or even as combination therapy for obesity or other disease processes.

## Additional file

Additional file 1: **Table S1.** Fatty acid pattern of the diets. **Table S2.** Primer sequences. **Table S3.** Primer Qiagen. **Table S4.** List of antibodies. **Table S5.** Correlation analysis data on the regulation of metabolic gene expression and UCP1 expression. **Table S6.** Correlation analysis data on genes involved in energy metabolism in iBAT. **Table S7.** Correlation analysis data on macrophage phenotype, its regulation and possible involvement in thermogenesis in iBAT. (PDF 129 kb)

## Abbreviations

ACC: Acetyl-CoA carboxylase
ACOX1: Acyl-CoA oxidase 1
ACTb: β-actin
ADRB: β-adrenergic receptor
AGPAT9: 1-acylglycerol-3-phosphate O-acyltransferase 9
AMPK: AMP-activated protein kinase
ATGL: Adipose triglyceride lipase
BAT: Brown adipose tissue
C: Control diet / group
CD36: Fatty acid translocase
CDH5: Cadherin 5
CLEC10a: C-type lectin domain family 10, member A (CD301)
CLS: Crown-like structures
CPT1a: Carnitine palmitoyltransferase 1a
CS: Citrate synthase
CYP4a10: Cytochrome P450, family 4, subfamily a, polypeptide 10
CYPB: Cyclophilin B
DGAT: Diglyceride acyltransferase
DHA: Docosahexaenoic acid
DIO: Diet-induced obesity
DIO2: Iodothyronine deiodinase 2
DIT: Diet-induced thermogenesis
DNL: de novo lipogenesis
EMR1: EGF-like module-containing mucin-like hormone receptor-like 1 (F4/80)
EPA: Eicosapentaenoic acid
FA: Fatty acid
FABP3: Fatty acid binding protein 3
FFA: Free fatty acids
FGF21: Fibroblast growth factor 21
G-3-P: Glycerol-3-phosphate
GAPDH: Glyceraldehyde-3-phosphate-dehydrogenase
GLUT: Glucose transporter
GPR120: G protein-coupled receptor 120
GYK: Glycerol kinase
HF: High-fat diet / group
HF/n-3: n-3 LCPUFA-enriched HFD / group
HFD: High-fat diet
HK2: Hexokinase 2
HPRT1: Hypoxanthine-guanine phosphoribosyltransferase 1
HSL: Hormone sensitive lipase
HSP90: Heat shock protein 90
HSP90αb1: Heat shock protein 90 (cytosolic) and class B member 1
iBAT: Interscapular brown adipose tissue
IL: Interleukin
ITGAX: Integrin αX (CD11c)
LCPUFA: Long-chain polyunsaturated fatty acids
LDHB: Lactate dehydrogenase
LEP: Leptin
LPBE: Enoyl-Coenzyme A hydratase
LPL: Lipoprotein lipase
LSI: Lower small intestine
M1: Type 1 macrophage
M2: Type 2 macrophage
MCL: cluster of macrophages
MCP1: Monocyte chemoattractant protein-1
MRC1: Mannose receptor, C type 1 (CD206)
MUFA: Monounsaturated fatty acid
NAD(P)H oxidase: Nicotinamide adenine dinucleotide (phosphate)-oxidase
NEFA: Non-esterified fatty acids
p47PHOX: Subunit of NAD(P)H oxidase
PGC1: Peroxisome proliferative activated receptor gamma coactivator 1
PPAR: Peroxisome proliferator activated receptor
PUFA: Polyunsaturated fatty acids
r: Pearson correlation coefficient
RT-qPCR: Real-time quantitative polymerase chain reaction
SEM: Standard error of the means
SFA: Saturated fatty acids
SIGLEC5: Sialic acid binding Ig-like lectin 5
TAG: Triacylglycerols
TGFβ: Transforming growth factor β
TGR5: G protein-coupled bile acid receptor
TH: Tyrosine hydroxylase
TLE3: Transducin-like enhancer of split 3
TNFα: Tumor necrosis factor α
UCP1: Uncoupling protein 1
USI: Upper small intestine
WAT: White adipose tissue

## References

[1] World Health Organization (2016). Obesity and overweight.

[2] Eckel RH, Grundy SM, Zimmet PZ (2005). The metabolic syndrome. Lancet.

[3] Cordain L, Eaton SB, Sebastian A, Mann N, Lindeberg S, Watkins BA (2005). Origins and evolution of the western diet: health implications for the 21st century. Am J Clin Nutr.

[4] Hammad SS, Jones PJ (2017). Dietary fatty acid composition modulates obesity and interacts with obesity-related genes. Lipids.

[5] Nettleton JA, Jebb S, Risérus U, Koletzko B, Fleming J (2014). Role of dietary fats in the prevention and treatment of the metabolic syndrome. Ann Nutr Metab.

[6] Simopoulos AP (2016). An increase in the omega-6/omega-3 fatty acid ratio increases the risk for obesity. Nutrients.

[7] Martínez-Fernández L, Laiglesia LM, Huerta AE, Martínez JA, Moreno-Aliaga MJ (2015). Omega-3 fatty acids and adipose tissue function in obesity and metabolic syndrome. Prostaglandins Other Lipid Mediat.

[8] Buckley JD, Howe PR (2009). Anti-obesity effects of long-chain omega-3 polyunsaturated fatty acids. Obes Rev.

[9] Titos E, Rius B, González-Périz A, López-Vicario C, Morán-Salvador E, Martínez-Clemente M (2011). Resolvin D1 and its precursor docosahexaenoic acid promote resolution of adipose tissue inflammation by eliciting macrophage polarization toward an M2-like phenotype. J Immunol.

[10] Hellmann J, Tang Y, Kosuri M, Bhatnagar A, Spite M (2011). Resolvin D1 decreases adipose tissue macrophage accumulation and improves insulin sensitivity in obese-diabetic mice. FASEB J.

[11] Flachs P, Horakova O, Brauner P, Rossmeisl M, Pecina P, Franssen-van Hal N (2005). Polyunsaturated fatty acids of marine origin upregulate mitochondrial biogenesis and induce beta-oxidation in white fat. Diabetologia.

[12] Janovská P, Flachs P, Kazdová L, Kopecký J (2013). Anti-obesity effect of n-3 polyunsaturated fatty acids in mice fed high-fat diet is independent of cold-induced thermogenesis. Physiol Res.

[13] Dossi CG, Tapia GS, Espinosa A, Videla LA, D’Espessailles A (2014). Reversal of high-fat diet-induced hepatic steatosis by n-3 LCPUFA: role of PPAR-α and SREBP-1c. J Nutr Biochem.

[14] van Schothorst EM, Flachs P, Franssen-van Hal NL, Kuda O, Bunschoten A, Molthoff J (2009). Induction of lipid oxidation by polyunsaturated fatty acids of marine origin in small intestine of mice fed a high-fat diet. BMC Genomics.

[15] Kim SH, Plutzky J (2016). Brown fat and Browning for the treatment of obesity and related metabolic disorders. Diabetes Metab J.

[16] Cannon B, Nedergaard J (2004). Brown adipose tissue: function and physiological significance. Physiol Rev.

[17] Villarroya F, Vidal-Puig A (2013). Beyond the sympathetic tone: the new brown fat activators. Cell Metab.

[18] Watanabe M, Houten SM, Mataki C, Christoffolete MA, Kim BW, Sato H (2006). Bile acids induce energy expenditure by promoting intracellular thyroid hormone activation. Nature.

[19] Nguyen KD, Qiu Y, Cui X, Goh YP, Mwangi J, David T (2011). Alternatively activated macrophages produce catecholamines to sustain adaptive thermogenesis. Nature.

[20] Bonet ML, Mercader J, Palou A (2017). A nutritional perspective on UCP1-dependent thermogenesis. Biochimie.

[21] Sadurskis A, Dicker A, Cannon B, Nedergaard J (1995). Polyunsaturated fatty acids recruit brown adipose tissue: increased UCP content and NST capacity. Am J Phys.

[22] Takahashi Y, Ide T (2000). Dietary n-3 fatty acids affect mRNA level of brown adipose tissue uncoupling protein 1, and white adipose tissue leptin and glucose transporter 4 in the rat. Br J Nutr.

[23] Bargut TC, Silva-e-Silva AC, Souza-Mello V, Mandarim-de-Lacerda CA, Aguila MB (2016). Mice fed fish oil diet and upregulation of brown adipose tissue thermogenic markers. Eur J Nutr.

[24] Sanderson LM, de Groot PJ, Hooiveld GJ, Koppen A, Kalkhoven E, Müller M (2008). Effect of synthetic dietary triglycerides: a novel research paradigm for nutrigenomics. PLoS One.

[25] Georgiadi A, Boekschoten MV, Müller M, Kersten S (2012). Detailed transcriptomics analysis of the effect of dietary fatty acids on gene expression in the heart. Physiol Genomics.

[26] Ludwig T, Worsch S, Heikenwalder M, Daniel H, Hauner H, Bader BL (2013). Metabolic and immunomodulatory effects of n-3 fatty acids are different in mesenteric and epididymal adipose tissue of diet-induced obese mice. Am J Physiol Endocrinol Metab.

[27] Livak KJ, Schmittgen TD (2001). Analysis of relative gene expression data using real-time quantitative PCR and the 2(−Delta Delta C (T)) method. Methods.

[28] Kotzbeck P, Giordano A, Mondini E, Murano I, Severi I, Venema W (2018). Brown adipose tissue whitening leads to brown adipocyte death and adipose tissue inflammation. J Lipid Res.

[29] Hardie DG (2011). Sensing of energy and nutrients by AMP-activated protein kinase. Am J Clin Nutr.

[30] Ventura-Clapier R, Garnier A, Veksler V (2008). Transcriptional control of mitochondrial biogenesis: the central role of PGC-1alpha. Cardiovasc Res.

[31] Nielsen TS, Jessen N, Jørgensen JO, Møller N, Lund S (2014). Dissecting adipose tissue lipolysis: molecular regulation and implications for metabolic disease. J Mol Endocrinol.

[32] Quesada-López T, Cereijo R, Turatsinze JV, Planavila A, Cairó M, Gavaldà-Navarro A (2016). The lipid sensor GPR120 promotes brown fat activation and FGF21 release from adipocytes. Nat Commun.

[33] Qiu Y, Nguyen KD, Odegaard JI, Cui X, Tian X, Locksley RM (2014). Eosinophils and type 2 cytokine signaling in macrophages orchestrate development of functional beige fat. Cell.

[34] Dinh CH, Szabo A, Yu Y, Camer D, Zhang Q, Wang H (2015). Bardoxolone methyl prevents fat deposition and inflammation in brown adipose tissue and enhances sympathetic activity in mice fed a high-fat diet. Nutrients.

[35] Jin L, Lin Z, Xu A (2016). Fibroblast growth factor 21 protects against atherosclerosis via fine-tuning the multiorgan crosstalk. Diabetes Metab J.

[36] Hondares E, Rosell M, Díaz-Delfín J, Olmos Y, Monsalve M, Iglesias R (2011). Peroxisome proliferator-activated receptor α (PPARα) induces PPARγ coactivator 1α (PGC-1α) gene expression and contributes to thermogenic activation of brown fat: involvement of PRDM16. J Biol Chem.

[37] Festuccia WT, Blanchard PG, Deshaies Y (2011). Control of brown adipose tissue glucose and lipid metabolism by PPARγ. Front Endocrinol.

[38] Villanueva CJ, Vergnes L, Wang J, Drew BG, Hong C, Tu Y (2013). Adipose subtype-selective recruitment of TLE3 or Prdm16 by PPARy specifies lipid storage versus thermogenic gene programs. Cell Metab.

[39] Chau MD, Gao J, Yang Q, Wu Z, Gromada J (2010). Fibroblast growth factor 21 regulates energy metabolism by activating the AMPK-SIRT1-PGC-1alpha pathway. Proc Natl Acad Sci U S A.

[40] Roberts-Toler C, O’Neill BT, Cypess AM (2015). Diet-induced obesity causes insulin resistance in mouse brown adipose tissue. Obesity.

[41] Fujisaka S, Usui I, Bukhari A, Ikutani M, Oya T, Kanatani Y (2009). Regulatory mechanisms for adipose tissue M1 and M2 macrophages in diet-induced obese mice. Diabetes.

[42] Fiamoncini J, Lima TM, Hirabara SM, Ecker J, Gorjão R, Romanatto T (2015). Medium-chain dicarboxylic acylcarnitines as markers of n-3 PUFA-induced peroxisomal oxidation of fatty acids. Mol Nutr Food Res.

[43] Lodhi IJ, Semenkovich CF (2014). Peroxisomes: a nexus for lipid metabolism and cellular signaling. Cell Metab.

[44] Wanders RJ, Waterham HR, Ferdinandusse S (2016). Metabolic interplay between peroxisomes and other subcellular organelles including mitochondria and the endoplasmic reticulum. Front Cell Dev Biol.

[45] Dahlhoff C, Worsch S, Sailer M, Hummel BA, Fiamoncini J, Uebel K (2014). Methyl-donor supplementation in obese mice prevents the progression of NAFLD, activates AMPK and decreases acyl-carnitine levels. Mol Metab.

[46] Ferramosca A, Conte A, Damiano F, Siculella L, Zara V (2014). Differential effects of high-carbohydrate and high-fat diets on hepatic lipogenesis in rats. Eur J Nutr.

[47] Coskun T, Bina HA, Schneider MA, Dunbar JD, Hu CC, Chen Y, Moller DE, Kharitonenkov A (2008). Fibroblast growth factor 21 corrects obesity in mice. Endocrinology.

[48] Mottillo EP, Desjardins EM, Fritzen AM, Zou VZ, Crane JD, Yabut JM, Kiens B, Erion DM, Lanba A, Granneman JG, Talukdar S, Steinberg GR (2017). FGF21 does not require adipocyte AMP-activated protein kinase (AMPK) or the phosphorylation of acetyl-CoA carboxylase (ACC) to mediate improvements in whole-body glucose homeostasis. Mol Metab.

[49] Flachs P, Rossmeisl M, Kuda O, Kopecky J (1831). Stimulation of mitochondrial oxidative capacity in white fat independent of UCP1: a key to lean phenotype. Biochim Biophys Acta.

[50] Sanders MA, Madoux F, Mladenovic L, Zhang H, Ye X, Angrish M (2015). Endogenous and synthetic ABHD5 ligands regulate ABHD5-Perilipin interactions and lipolysis in fat and muscle. Cell Metab.

[51] Vergnes L, Chin R, Young SG, Reue K (2011). Heart-type fatty acid-binding protein is essential for efficient brown adipose tissue fatty acid oxidation and cold tolerance. J Biol Chem.

[52] Mazzucotelli A, Viguerie N, Tiraby C, Annicotte JS, Mairal A, Klimcakova E (2007). The transcriptional coactivator peroxisome proliferator activated receptor (PPAR) gamma coactivator-1 alpha and the nuclear receptor PPAR alpha control the expression of glycerol kinase and metabolism genes independently of PPAR gamma activation in human white adipocytes. Diabetes.

[53] La Rovere MT, Christensen JH (2015). The autonomic nervous system and cardiovascular disease: role of n-3 PUFAs. Vasc Pharmacol.

[54] Fischer K, Ruiz HH, Jhun K, Finan B, Oberlin DJ, van der Heide V (2017). Alternatively activated macrophages do not synthesize catecholamines or contribute to adipose tissue adaptive thermogenesis. Nat Med.

[55] Oh DY, Talukdar S, Bae EJ, Imamura T, Morinaga H, Fan W (2010). GPR120 is an omega-3 fatty acid receptor mediating potent anti-inflammatory and insulin-sensitizing effects. Cell.

[56] Prieur X, Lesnik P, Moreau M, Rodríguez JC, Doucet C, Chapman MJ (2009). Differential regulation of the human versus the mouse apolipoprotein AV gene by PPARalpha: Implications for the study of pharmaceutical modifiers of hypertriglyceridemia in mice. Biochim Biophys Acta.

[57] Rakhshandehroo M, Hooiveld G, Müller M, Kersten S (2009). Comparative analysis of gene regulation by the transcription factor PPARalpha between mouse and human. PLoS One.

[58] Krishnan S, Cooper JA (2014). Effect of dietary fatty acid composition on substrate utilization and body weight maintenance in humans. Eur J Nutr.

[59] Aranceta J, Pérez-Rodrigo C (2012). Recommended dietary reference intakes, nutritional goals and dietary guidelines for fat and fatty acids: a systematic review. Recommended dietary reference intakes, nutritional goals and dietary guidelines for fat and fatty acids: a systematic review. Br J Nutr.

[60] Wolfram G, Bechthold A, Boeing H, Ellinger S, Hauner H, Kroke A (2015). German nutrition society. Evidence-based guideline of the German nutrition society: fat intake and prevention of selected nutrition-related diseases. Ann Nutr Metab.

[61] Fan R, Koehler K, Chung S. Adaptive thermogenesis by dietary n-3 polyunsaturated fatty acids: emerging evidence and mechanisms. Biochim Biophys Acta. 2018. 10.1016/j.bbalip.2018.04.012. 10.1016/j.bbalip.2018.04.012 PMC6197923 29679742

[62] Illig T, Gieger C, Zhai G, Römisch-Margl W, Wang-Sattler R, Prehn C (2010). A genome-wide perspective of genetic variation in human metabolism. Nat Genet.

