# Supplementary material for: Dietary n-3 long-chain polyunsaturated fatty acids upregulate energy dissipating metabolic pathways conveying anti-obesogenic effects in mice
Source: Nutr Metab (Lond). 2018 Sep 26;15:65. doi: 10.1186/s12986-018-0291-x (PMC6158869; doi:10.1186/s12986-018-0291-x)
Supplement: Supplementary file 1 — Table S1. Fatty acid pattern of the diets. Table S2. Primer sequences. Table S3. Primer Qiagen. Table S4. List of antibodies. Table S5. Correlation analysis data on the regulation of metabolic gene expression and UCP1 expression. Table S6. Correlation analysis data on genes involved in energy metabolism in iBAT. Table S7. Correlation analysis data on macrophage phenotype, its regulation and possible involvement in thermogenesis in iBAT. (PDF 129 kb) [file 12986_2018_291_MOESM1_ESM.pdf]

Worsch et al, 2018

Additional file 1:

Table S1 Fatty acid pattern of the diets

Fatty acid species			Weight%		
			C	HF	HF/n-3
Lauric acid	12:0		0.07	0.13	0.09
Myristic acid	14:0		0.30	0.88	0.66
Pentadecanoic acid	15:0		0.04	0.05	0.07
Palmitic acid	16:0		11.37	36.70	23.91
Palmitoleic acid	16:1		0.12	0.15	0.30
Heptadecanoic acid	17:0		0.09	0.10	0.18
Heptadecenoic acid	17:1		0.05	-	0.07
Stearic acid	18:0		3.60	4.18	4.00
Elaidic acid	18:1		0.08	0.14	0.07
Oleic acid	18:1		23.47	35.81	26.08
cis-Vaccenic acid	18:1		1.53	0.87	1.11
18:2 Isomer	18:2		0.38	0.20	0.14
Linoleic acid	18:2	n-6	50.85	18.07	16.00
y-Linolenic acid	18:3	n-6	-	-	0.12
Conjugated Linoleic acid	18:2		0.11	-	-
18:3 Isomer	18:3		0.39	0.09	0.10
Linolenic acid	18:3	n-3	5.16	1.34	1.40
Stearidonic acid	18:4	n-3	-	-	0.16
Arachidic acid	20:0		0.35	0.38	0.48
Eicosenoic acid	20:1		0.22	0.15	0.83
Eicosadienoic acid	20:2	n-6	-	-	0.21
Heneicosanoic acid	21:0		-	-	0.04
Eicosatrienoic acid	20:3	n-6	-	-	0.09
Arachidonic acid	20:4	n-6	-	-	0.90
Eicosatrienoic acid	20:3	n-3	-	-	0.14
Eicosapentaenoic acid (EPA)	20:5	n-3	-	-	4.05
Behenic acid	22:0		0.48	0.16	0.27
Cetoleic acid	22:1		0.11	-	0.11
Erucic acid	22:1		-	-	0.12
Docosatetraenoic acid	22:4	n-6	-	-	0.11
Docosapentaenoic acid	22:5	n-6	-	-	0.77
Docosapentaenoic acid	22:5	n-3	-	-	0.88
Docosahexaenoic acid (DHA)	22:6	n-3	-	-	15.05
Tricosanoic acid	23:0		0.04	-	-
Lignoceric acid	24:0		0.17	0.09	0.13
Nervonic acid	24:1		0.36	-	0.36

Table S1 continued

	C	HF	HF/n-3	Refefernce to Human diet*
Metabolizable energy, fat and fatty acids (kJ%)	13	48	48	35
Σ SFA	2.2	20.5	14.3	10
Σ MUFA	3.4	17.8	14.0	15-20
Σ PUFA	3.6	9.5	19.3	6-11
Σ n-6 PUFA	3.2	8.7	8.7	2.5-9
Σ n-3 PUFA	0.3	0.6	10.4	0.5-2

Fatty acids from different diets were analysed by gas chromatography and expressed as percentage by weight of total fatty acids (weight%). C, control diet (Cat. no.: S5745-E720); HF, high-fat diet (Cat. no.: S5745-E722); HF/n-3, n-3 long-chain polyunsaturated fatty acid-enriched high-fat diet (Cat. no.: S5745-E725). Diets were manufactured by Ssniff Spezialdiäten (Soest, Germany). To compare kJ% of fat or fatty acid classes from the diets of the mouse study with data for dietary recommendations for human, *data for dietary recommendations of the FAO/WHO (see Table 1 in Aranceta J and Pérez-Rodrigo C,(2012),.

*Aranceta J, Pérez-Rodrigo C. Recommended dietary reference intakes, nutritional goals and dietary guidelines for fat and fatty acids: a systematic review. Br J Nutr. 2012 Jun;107 Suppl 2:S8-22.

Table S2 Primer sequences

Target Gene		Primer 5'-3'	Target Gene		Primer 5'-3'
Adrb1	fw	CGT CCG TCG TCT CCT TCT AC	Gyk	fw	CAA ATG CAA GCA GGA CGA TG
	rev	CAT GAT GAT GCC CAG TGT CTT G		rev	AGG CCC CAG CTT TCA TTA GG
Adrb3	fw	CAG CCA GCC CTG TTG AAG	Hk2	fw	AGA GAA CAA GGG CGA GGA GC
	rev	CCT TCA TAG CCA TCA AAC CTG		rev	GGA GGA AGC GGA CAT CAC AAT
Acc2	fw	AGG GTC ATA GAG AAG GTG CTC A	Hprt1	fw	GTC GTG ATT AGC GAT GAT GAA CC
	rev	AGA TCC TCG GGC GTC ACC AT		rev	GTC TTT CAG TCC TGT CCA TAA TCA G
Acox1	fw	GAG ATG GAT AAT GGC TAC CTG AAG	Hsl	fw	GAA CTA AGT GGA CGC AAG CC
	rev	AAA CCA TGG TCC CAT ATG TCA GC		rev	TTG ACA TCA GAG GGT GTG GA
Actb	fw	CCA CTG CCG CAT CCT CTT CC	Hsp90αb1	fw	AGG AGG GTC AAG GAA GTG GT
	rev	GCC ACA GGA TTC CAT ACC CAA GA		rev	TTT TTC TTG TCT TTG CCG CT
CD36	fw	CTT GAG AAG ACA ATC AAA AGG GAA G	Itgax	fw	GCA GGA GTG TCC AAA GCA AGA C
	rev	GTC CTC GGG GTC CTG AGT TAT		rev	CTG AAG CTG GCT CAT CAC AGC
Cdh5	fw	CCC ACT ATG TGG GAA AGA TCA AGT	Ldhb	fw	ATG GTG AAG GGA ATG TAC GGC
	rev	ATG AGG GCA GTA AGG AAG TAC TCA		rev	GCT TCT GAT TGA TGA CGC TGG T
Cpt1a	fw	GTC CCA GCT GTC AAA GAT ACC G	Lep	fw	ACA TTT CAC ACA CGC AGT CGG
	rev	ATG GCG TAG TAG TTG CTG TTA ACC		rev	AGG CAG GCT GGT GAG GAC CT
Cs	fw	CTG AGG AAG ACT GAC CCT CG	Lpbe	fw	GCT TGC CCA ACA TGG ACA GTG
	rev	TTC ATC TCC GTC ATG CCA TA		rev	GCC TGG ACT GAA CGG ACA CAA G
Cyp4a10	fw	CTA AGC CCA ACC CGA TTT GC	Mcp1	fw	GCT CAG CCA GAT GCA GTT AAC G
	rev	TTG CCT GTG GAG GTA GAA CTG G		rev	GCT TGG TGA CAA AGA CTA CAG CTT
CypB	fw	TCG TCT TTG GAC TCT TTG GAA	Mrc1	fw	GCC AGG ACG AAA GGC GGG AT
	rev	TCC TTG ATG ACA CGA TGG AA		rev	GGA GTT GTT GTG GGC TCT GGT G
Dgat1	fw	TCC AGA CAA CCT GAC CTA CCG	p47phox	fw	CGG CAC CCA GGT GGT TTG AT
	rev	ACC ATC CAC TGT TGG ATC AGC		rev	TCA GTG GGC AGT TTC AGG TCA T
Dio2	fw	ACA GGT TAA ACT GGG TGA AGA TGC	Pgc1α	fw	GGA CGG AAG CAA TTT TTC AA
	rev	CAG TTG CCT AGT GAA AGG TGG T		rev	GAG TCT TGG GAA AGG ACA CG
Fabp3	fw	ATC CAT GTG CAG AAG TGG AA	Pgc1β	fw	CGA GCT CTT CCA GAT TGA CAG T
	rev	CAC TGC CAT GAG TGA GAG TCA		rev	TGC AGG ATG GTG TGT CGC CTT
Fgf21	fw	TGG AAT GGA TGA GAT CTA GAG TTG G	Pparα	fw	CCA GTA CTT AGG AAG CTG TCC G
	rev	GAG CTC CAG GAG ACT TTC TGG A		rev	TAT TCG ACA CTC GAT GTT CAG GG
Gapdh	fw	CCT GGA GAA ACC TGC CAA GTA TG	Pparγ2	fw	ACT CTG GGA GAT TCT CCT GTT GTC
	rev	GAG TGG GAG TTG CTG TTG AAG TC		rev	CAT GGT GGT TTC TTG TGA AGT GCT
Glut1	fw	CGT CAG GGC GTG GAG GTC	Tgr5	fw	CAG GAG GCC ATA AAC TTC CA
	rev	CAC CTT CTT GCT GCT GGG ATC		rev	GTC AGC TCC CTG TTC TTT G
Glut4	fw	TGT TGC GGA TGC TAT GGG TCC T	Tle3	fw	TGG TGA GCT TTG GAG CTG TT
	rev	CAC CTC CTG CTC TAA AAG GGA AG		rev	CGG TTT CCC TCC AGG AAT
Gpr120	fw	CGG CGG GGA CCA GGA AAT TC			
	rev	ACC AGT CCC GGC ACC AGG A			

Table S3 Primer Qiagen

Target Gene	Primer	Target Gene	Primer
Acc1	order no QT01554441	Lpl	order no QT01750469
Clec10a	order no QT00151011	Siglec5	order no QT00297556
Dgat2	order no QT00134477	Tgfβ1	order no QT00145250
Emr1	order no QT00099617	Tnfα	order no QT00104006
IL10	order no QT00106169		

Table S4 List of antibodies

Antibody	Application	Description	Company
ACC1/2	WB; 1:1000	monoclonal, rabbit anti-mouse	3676, cell signaling/New England Biolabs, Frankfurt am Main, Germany
AMPKα	WB; 1:1000	polyclonal, rabbit anti-mouse	2532, cell signaling/New England Biolabs, Frankfurt am Main, Germany
ATGL	WB; 1:1000	monoclonal, rabbit anti-mouse	2138, cell signaling/New England Biolabs, Frankfurt am Main, Germany
β-ACTIN	WB; 1:1000	polyclonal, rabbit anti-mouse	4967, cell signaling/New England Biolabs, Frankfurt am Main, Germany
CD11c	IHC-P; 1:5000	monoclonal, hamster anti-mouse	553799, BD Biosciences, Heidelberg, Germany
CD206	IHC-P; 1:1500	polyclonal, rabbit anti-human	18704-1-AP, Proteintech, Chicago, IL, USA
CD301	IHC-P; 1:40	monoclonal, rat anti-mouse	LS-C123200, LifeSpan Biosciences, Seattle, WA, USA
CS	WB; 1:5000	polyclonal, rabbit anti-mouse	ab96600, Abcam, Cambridge, UK
F4/80	IHC-P; 1:120	monoclonal, rat anti-mouse	T-2006, BMA Biomedicals, Augst, Switzerland
HSL	WB; 1:1000	polyclonal, rabbit anti-mouse	4107, cell signaling/New England Biolabs, Frankfurt am Main, Germany
HSP90	WB; 1:1,000	monoclonal, mouse anti-mouse	SMC-149, StressMarq Biosciences Inc, Cadboro Bay, Victoria, Canada
phospho-ACC1 (Ser79)	WB; 1:500	polyclonal, rabbit anti-mouse	07-303, merck millipore, Billerica, MA, USA
phospho-ACC2 (Ser219/Ser221)	WB; 1:500	polyclonal, rabbit anti-mouse	sc-30446, santa cruz, Heidelberg, Germany
phospho-AMPKα (Thr172)	WB; 1:500	monoclonal, rabbit anti-mouse	2535, cell signaling/New England Biolabs, Frankfurt am Main, Germany
phospho-HSL	WB;	polyclonal, rabbit anti-mouse	4126, cell signaling/New England Biolabs,

(Ser660)	1:1000		Frankfurt am Main, Germany
phospho-HSL (Ser563)	WB; 1:1000	polyclonal, rabbit anti-mouse	4139, cell signaling/New England Biolabs, Frankfurt am Main, Germany
phospho-HSL (Ser565)	WB; 1:1000	polyclonal, rabbit anti-mouse	4137, cell signaling/New England Biolabs, Frankfurt am Main, Germany
TH	WB; 1:1000	polyclonal, rabbit anti-mouse	AB152, merck millipore, Billerica, MA, USA
UCP1	WB; 1:10000	serum, rabbit anti-hamster	Klingenspor et al. [1]
2° antibody	WB; 1:20000	IRDye800CW-conjugated goat anti-rabbit IgG (H+L)	926-32211, LI-COR Biosciences, Bad Homburg, Germany
2° antibody	WB; 1:20000	IRDye680RD-conjugated donkey anti-mouse IgG (H+L)	925-68072, LI-COR Biosciences, Bad Homburg, Germany
2° antibody	IHC-P; 1:1000	rabbit anti-rat IgG	312-005-045, Jackson ImmunoResearch, West Grove, PA, USA
2° antibody	IHC-P; 1:1000	rabbit anti-Syrian Hamster IgG	NB120-6699, Novus Biologicals, Littleton, CO, USA

WB, Western blot; IHC-P, immunohistochemistry-paraffin.

Table S5 Correlation analysis data on the regulation of metabolic gene expression and UCP1 expression

Gene	Gene / Protein (WB)		Correlation			
			Total	C	HF	HF/n-3
Pparα	Ucp1#	p	0.0006***	0.6483	0.9713	0.0795
		r	0.5653	-0.1553	0.01167	0.5789
Gpr120	Ucp1#	p	0.0013**	0.2812	0.1563	0.0203*
		r	0.5424	-0.3782	0.4362	0.7144
Fgf21	Ucp1#	p	<0.0001***	0.4879	0.0243*	0.0185*
		r	0.6331	0.2221	0.6422	0.7214
Tle3	Ucp1#	p	0.0487*	0.5308	0.0474*	0.3041
		r	0.3406	-0.2011	-0.5814	-0.3619

Shown are correlations between key metabolic regulators and UCP1 in the interscapular brown adipose tissue (iBAT) after 12-week feeding either control (C), HFD (HF), or n-3 long-chain polyunsaturated fatty acid (LCPUFA)-enriched HFD (HF/n-3). The expression data were generated by RT-qPCR (n = 8-12) and correlation analyses were performed utilizing the whole data set (total), but also analysing the data from each group separately. For the genes marked with a hash symbol, the data were obtained from Ludwig et al. [2]. *p < 0.05, **p < 0.01, ***p < 0.001, indicate significant correlation. p, statistical significance; r, Pearson correlation coefficient.

Table S6 Correlation analysis data on genes involved in energy metabolism in iBAT

A FGF21 as regulator of possible key proteins involved in energy metabolism in iBAT

Gene	Gene		Correlation			
			**Total**	**C**	**HF**	**HF/n-3**
Fgf21	Adrb1	p	0.0004***	0.0198*	0.0055**	0.0016**
		r	0.5759	0.6590	0.7443	0.8557
Fgf21	CD36	p	<0.0001***	0.0224*	0.0111*	0.0320*
		r	0.7477	0.6490	0.7011	0.6756
Fgf21	Dgat1	p	0.0071**	0.0015*	0.0036**	0.0462*
		r	0.4664	0.8066	0.7927	0.6746
Adrb1	Dgat1	p	0.0010***	0.0055**	0.0739	0.0099**
		r	0.5552	0.7443	0.5589	0.7982
Adrb1	CD36	p	0.0044**	0.0795	0.0502	0.0112*
		r	0.4762	0.5252	0.5756	0.7573
Dgat1	CD36	p	0.0157*	0.0031**	0.0869	0.0214*
		r	0.4235	0.7745	0.5393	0.7443

(**A, B**) Table S6B is shown on the next page. Shown are correlations between targets involved in energy metabolism in the interscapular brown adipose tissue (iBAT) after 12-week feeding either control (C), HFD (HF), or n-3 long-chain polyunsaturated fatty acid (LCPUFA)-enriched HFD (HF/n-3). The expression data were generated by RT-qPCR (n = 8-12) and correlation analyses were performed utilizing the whole data set (total), but also analysing the data from each group separately. For the genes marked with a hash symbol, the data were obtained from Ludwig et al [2]. *p < 0.05, **p < 0.01, ***p < 0.001, indicate significant correlation. p, statistical significance; r, Pearson correlation coefficient.

B Correlations between mRNA levels of Adrb1, Dgat1 and CD36 and proteins involved in energy metabolism

Gene	Gene		Correlation				Gene		Correlation				Gene		Correlation			
			Total	C	HF	HF/n-3			Total	C	HF	HF/n-3			Total	C	HF	HF/n-3
Ucp1[#]	Adrb1	p	0.1306	0.0794	0.0632	0.0558	CD36	p	0.0002***	0.2802	0.1128	0.1929	Dgat1	p	0.1000	0.1575	0.0657	0.0951
		r	0.2645	0.5253	0.5512	0.6201		r	0.6002	0.3396	0.4817	0.4491		r	0.2960	0.4350	0.5725	0.5890
Cs	Adrb1	p	0.0158*	0.0283*	0.0200*	0.0237*	CD36	p	<0.0001***	0.7453	0.2648	0.0174*	Dgat1	p	0.0161*	0.0347*	0.0487*	0.0012**
		r	0.4169	0.6562	0.6579	0.7018		r	0.6659	0.1110	0.3500	0.7264		r	0.4289	0.6379	0.6049	0.8920
Lpl	Adrb1	p	0.0366*	0.0393*	0.1376	0.0419*	CD36	p	<0.0001***	0.1624	<0.0001***	0.0459*	Dgat1	p	0.0110*	0.0874	0.2766	0.0193*
		r	0.3599	0.5998	0.4546	0.6499		r	0.7063	0.4305	0.9289	0.6409		r	0.4435	0.5140	0.3601	0.7527
Angptl4	Adrb1	p	0.0008***	0.1161	0.0113*	0.0294*	CD36	p	0.5869	0.1301	0.0795	0.1046	Dgat1	p	0.0023**	0.4867	0.1034	0.0094**
		r	-0.5616	-0.5014	-0.7265	-0.6833		r	-0.09977	-0.4855	-0.5502	-0.5432		r	-0.5348	-0.2350	-0.5448	-0.8015
Agpat9	Adrb1	p	0.0083**	0.0011**	0.0135*	0.1225	CD36	p	<0.0001***	0.1003	0.0327*	0.0174*	Dgat1	p	0.0592	0.0208*	0.1752	0.0042**
		r	0.4451	0.8193	0.6872	0.5211		r	0.6894	0.4969	0.6166	0.7263		r	0.3371	0.6548	0.4404	0.8439
Dgat2	Adrb1	p	0.0165*	0.0657	0.0014**	0.0627	CD36	p	<0.0001***	0.5016	0.0313*	0.0197*	Dgat1	p	0.0642	0.2048	0.0788	0.0013**
		r	0.4082	0.5470	0.8107	0.6071		r	0.6783	0.2153	0.6206	0.7166		r	0.3310	0.3942	0.5512	0.8898
Gyk	Adrb1	p	0.6042	0.0849	0.9165	0.0374*	CD36	p	<0.0001***	0.1517	0.0401*	0.0260*	Dgat1	p	0.2282	0.0985	0.9618	0.0009***
		r	0.09364	0.5175	0.03591	0.6610		r	0.6760	0.4406	0.6242	0.6938		r	0.2229	0.4992	0.01745	0.9013
Ldhb	Adrb1	p	0.0020**	0.0319*	0.1382	0.0159*	CD36	p	<0.0001***	0.0030**	0.3010	0.0018**	Dgat1	p	<0.0001***	<0.0001***	0.0011**	0.0001***
		r	0.5329	0.6190	0.5032	0.7670		r	0.6760	0.7753	0.3641	0.8782		r	0.8520	0.8930	0.8695	0.9622
Pck1	Adrb1	p	<0.0001***	0.0014**	0.0045**	0.0084**	CD36	p	0.0068**	0.3173	0.1112	0.0108*	Dgat1	p	0.0015**	0.0312*	0.1722	0.0449*
		r	0.6532	0.8108	0.7557	0.7753		r	0.4556	0.3158	0.4836	0.7595		r	0.5390	0.6210	0.4432	0.6777
Hsl	Adrb1	p	<0.0001***	0.0391*	0.0062**	0.0427*	CD36	p	0.0343*	0.3665	0.2520	0.0534	Dgat1	p	0.0109*	0.1347	0.0424*	0.1592
		r	0.6409	0.6267	0.7370	0.6481		r	0.3695	0.3021	0.3588	0.6248		r	0.4509	0.4805	0.6188	0.5115
Fabp3	Adrb1	p	0.3100	0.0112*	0.0046**	0.0557	CD36	p	<0.0001***	0.0757	0.0124*	0.0482*	Dgat1	p	0.6888	0.0173*	0.0710	0.0173*
		r	0.1794	0.7005	0.7541	0.6203		r	0.6589	0.5309	0.6934	0.6703		r	0.07363	0.6691	0.5636	0.7607
Cpt1a	Adrb1	p	0.0257*	0.0136*	0.0034**	0.0158*	CD36	p	<0.0001***	0.7314	0.0196*	0.0234*	Dgat1	p	0.6233	0.4382	0.1913	0.0022**
		r	0.3822	0.6870	0.7696	0.7332		r	0.6469	0.1109	0.6596	0.7027		r	0.09024	0.2474	0.4261	0.8718
Acc2	Adrb1	p	0.0189*	0.2081	0.2048	0.0592	CD36	p	0.1931	0.9954	0.9558	0.0572	Dgat1	p	0.2156	0.7477	0.6812	0.2047
		r	0.4004	0.3916	0.3942	0.6136		r	-0.2288	-0.001879	0.01798	0.6174		r	0.2251	0.1040	-0.1401	0.4673
Glut1	Adrb1	p	0.0084**	0.0142*	0.5073	0.0190*	CD36	p	0.0229*	0.0140*	0.4438	0.0043**	Dgat1	p	<0.0001***	0.0019**	0.9984	0.0406*
		r	0.4446	0.6840	-0.2125	0.7196		r	0.3892	0.6849	0.2445	0.8120		r	0.6446	0.7973	0.0006898	0.6876
Glut4	Adrb1	p	0.1283	0.8344	0.0668	0.3007	CD36	p	0.1635	0.5877	0.3209	0.0481*	Dgat1	p	0.0542	0.8567	0.0109*	0.3074
		r	0.2702	0.07155	0.5451	0.3643		r	-0.2483	0.1842	0.3136	0.6359		r	0.3492	0.06184	0.7290	0.3841
Hk2	Adrb1	p	0.0150*	0.5695	0.0194*	0.0308*	CD36	p	0.2496	0.0245*	0.0004***	0.0018**	Dgat1	p	0.0064**	0.2590	0.0351*	0.0018**
		r	0.4138	0.1828	0.6606	0.6792		r	0.2030	0.6415	0.8559	0.8498		r	0.4722	0.3540	0.6368	0.8788

Table S7 Correlation analysis data on macrophage phenotype, its regulation and possible involvement in thermogenesis in iBAT

Gene	Gene		Correlation			
			Total	C	HF	HF/n-3
Siglec5	Emr1	p	<0.0001***	0.8431	0.0694	0.0052**
		r	0.6916	-0.06412	0.5661	0.8027
Siglec5	Mrc1	p	<0.0001***	0.6795	0.0880	0.0225*
		r	0.6480	0.1333	0.5131	0.7059
Siglec5	Clec10a	p	0.0003***	0.0826	0.6175	0.0085**
		r	0.5779	0.5207	0.1608	0.7750
Siglec5	Ucp1[#]	p	0.0049**	0.1271	0.8127	0.0319*
		r	0.4717	-0.4657	-0.0767	0.6761
Gpr120	Emr1	p	0.1441	0.0181*	0.5595	0.0443*
		r	0.2686	-0.7231	-0.1980	0.6444
Gpr120	Clec10a	p	0.6638	0.5362	0.1437	0.0040**
		r	0.07990	-0.2227	-0.4484	0.8155
Gpr120	Siglec5	p	0.0070**	0.6072	0.3718	0.0404*
		r	0.4674	0.1859	-0.2835	0.6537

Shown are correlations between gene expression data of immune cell markers, and between metabolic markers or Gpr120, a target related to the regulation of inflammation, and immune cell markers in interscapular brown adipose tissue (iBAT) after 12-week feeding either control (C), HFD (HF), or n-3 long-chain polyunsaturated fatty acid (LCPUFA)-enriched HFD (HF/n-3). The expression data were generated by RT-qPCR analysis (n = 7-12) and correlation analyses were performed utilizing the whole data set (total), but also analysing the data from each group separately. For the genes marked with a hash symbol, the data were obtained from Ludwig et al. [2]. *p < 0.05, **p < 0.01, ***p < 0.001, indicate significant correlation. p, statistical significance; r, Pearson correlation coefficient.

References

1. Klingenspor M, Ebbinghaus C, Hülshorst G, Stöhr S, Spiegelhalter F, Haas K, et al. Multiple regulatory steps are involved in the control of lipoprotein lipase activity in brown adipose tissue. J Lipid Res. 1996;37:1685-95.
2. Ludwig T, Worsch S, Heikenwalder M, Daniel H, Hauner H, Bader BL. Metabolic and immunomodulatory effects of n-3 fatty acids are different in mesenteric and epididymal adipose tissue of diet-induced obese mice. Am J Physiol Endocrinol Metab. 2013;304:1140-56.
